# Effects of continuous cropping on soil metabolomics and rhizosphere bacterial communities in *Panax quinquefolius* L.

**DOI:** 10.3389/fmicb.2025.1698779

**Published:** 2025-11-26

**Authors:** Jian Song, Qing He, Kuo Cao, Xiliang Song, Qiangcheng Zeng

**Affiliations:** College of Life Sciences, Dezhou University, De’zhou, China

**Keywords:** continuous cropping obstacle, soil health, microbial ecology, metabolite profiling, *Panax quinquefolius* L.

## Abstract

**Introduction:**

Continuous cropping obstacles (CCOs) due to long-term monoculture have emerged as a pervasive challenge in contemporary agriculture worldwide. The practices of CCOs are the primary causes of restraining the *Panax quinquefolius* L. (*P. quinquefolius*) growth, whereas its underlying microbial mechanisms have not been fully elucidated.

**Methods:**

We investigated the effects of CCOs on soil physicochemical properties, enzyme activities, microbial community composition, and metabolite profiles in the rhizosphere of *P. quinquefolius* cultivated continuously for one, two, three, and four consecutive years (designated as CC1, CC2, CC3, and CC4, respectively) without crop rotation. Rhizosphere soil samples were collected from fields with different years of CCOs and analyzed for physicochemical properties and enzyme activities. Microbial community composition was assessed using Illumina high-throughput sequencing, and metabolite profiles were analyzed using non-targeted metabolomics (UPLC-MS/MS).

**Results:**

Significant decreases were observed in soil pH (12.2–28.0%), cation exchange capacity (42.6–65.5%), organic matter (8.7–27.3%), total nitrogen (7.6–27.8%), and ammonium (NH_4_^+^) content (16.9–56.6%) with an increasing number of continuous cropping years. Enzymatic activities, including urease, invertase, alkaline phosphatase, catalase, protease, and polyphenol oxidase, were also reduced. The occurrence of CCOs decreased bacterial richness and number but increased bacterial diversity. Key microbial biomarkers were shifted from Gemmatimonadota, Actinobacteriota, and Proteobacteria to Acidobacteriota, Chloroflexi, and WPS-2 with *P. quinquefolius* CCOs. Consequently, the number of beneficial microorganisms decreased, whereas the number of pathogenic microorganisms increased. Non-targeted metabolomic profiling showed significant enantioselectivity in phenylpropanoid biosynthesis and pyrimidine metabolism. Time-series analysis revealed a decrease in metabolites classified as lipids and lipid-like molecules and an increase in organic acids, derivatives, phenylpropanoids, and polyketides with continuous cropping. Partial least squares-path modeling identified reduced soil enzymatic activity due to CCOs as the primary factor regulating soil bacterial communities and metabolites.

**Significance:**

These findings offer new insights into the microecological mechanisms of CCOs in *P. quinquefolius*, aiding in controlling pathogenic bacteria and maintaining soil health in agricultural systems.

**Conclusion and prospects:**

*P. quinquefolius* CCOs significantly alter soil physicochemical properties, microbial community structure, and metabolite profiles, leading to reduced soil fertility and increased prevalence of soil-borne diseases. Future research should focus on exploring sustainable agricultural practices, such as crop rotation and soil amendments, to mitigate these adverse effects and improve the long-term viability of *P. quinquefolius* cultivation.

## 1 Introduction

Continuous cropping (CC) refers to repeated cultivation of the same crop in the same field over many years. Owing to limited arable land and rising food demand, CC remains a prevalent agricultural practice in developing countries (Lei et al., 2020; Tan et al., 2021). However, it places significant stress on the soil, leading to the degradation of soil properties (Pascale et al., 2020) and disruption in microbial communities (Beretta-Blanco et al., 2019). These challenges increase the risk of soil-borne diseases, deteriorate microbial structures, and impair crop productivity and quality (Chen et al., 2022a,b; She et al., 2017). Over the past few decades, significant progress has been made in recent decades in understanding the mechanisms underlying CC obstacles (CCOs) in crops such as *Nicotiana tabacum* (Chen et al., 2023), *Citrullus lanatus* (Ding et al., 2021), *Glycine max* (Cui et al., 2024), and *Panax notoginseng* (Wang F. et al., 2022), with a focus on soil health, plant nutrition, microbiology, and agricultural practices. These studies have revealed that the primary soil mechanisms causing CCOs include: (i) secretion of autotoxins from plant root exudates into the soil, leading to allelopathic autotoxicity (Zeeshan Ul Haq et al., 2023); (ii) degradation of soil’s physicochemical properties (Song et al., 2018; Wang F. et al., 2022); and (iii) disruption of soil microbial biomass, activity, and community structure (Ashworth et al., 2020; Zhao et al., 2024). These factors lead to plant growth inhibition and soil-borne diseases (Li H. et al., 2019), ultimately affecting crop yield. Understanding the mechanisms underlying CC-induced obstacles is critical to maintaining soil fertility and ensuring sustainable food production.

Soil microorganisms are essential for biogeochemical cycles and energy transfer (Rillig et al., 2019; Xue et al., 2022). Several studies have shown that disturbances in soil microecology and proliferation of pathogenic microorganisms are primary contributors to CCOs (Zhang H. et al., 2022). The CCOs lowers soil pH and suppresses the abundance of beneficial bacteria, including *Sphingomonas*, *Bacillus*, and *Prevotella* (Arafat et al., 2020; Li J. et al., 2023). For instance, continuous tobacco cultivation significantly reduced soil organic matter (SOM), available phosphorus (AP), total nitrogen (TN), and nitrate (NO_3_^–^), negatively affecting microbial diversity and community structure (Tan et al., 2021). Furthermore, long-term CCOs diminish soil fertility (Tan et al., 2017), inhibit soil enzyme activity (Li J. et al., 2023), disrupt the microbial balance (Ma et al., 2023), degrade the soil microecological environment (Liao and Xia, 2024), and facilitate the spread of soil-borne pathogens (Chen et al., 2018). These effects increase the prevalence of diseases such as blight and *Verticillium* wilt (Li J. et al., 2023; Tan et al., 2017), which exacerbate plant health challenges and crop productivity. The rhizosphere microbiome plays a crucial role in maintaining plant and soil health by suppressing disease development (de Faria et al., 2021; Trivedi et al., 2021). Complex microbial interactions within the rhizosphere influence the susceptibility of plants to infections and affect their growth (Semenov et al., 2020). Therefore, monitoring the alterations in rhizosphere microbial communities is essential for identifying the root causes of CCOs-induced challenges and developing strategies to enhance plant disease resistance.

In addition to microbial disturbances, CCOs also affect soil metabolite composition and metabolic processes. Changes in soil metabolites, indicative of the physiological and metabolic activities of microbes, offer crucial insights into microbial responses to environmental stress (Cui et al., 2024; Dong et al., 2024; Gong et al., 2023). Metabolomics, a powerful technique for the qualitative and quantitative analysis of low-molecular-weight metabolites, provides a deeper understanding of the biological processes and functional capabilities of microbes (Muthubharathi et al., 2021). This technique has been widely used to monitor soil metabolic responses to stressors, such as salinization (Duan et al., 2023), organic pollutants (Phulpoto et al., 2024), heavy metals (Feng et al., 2024), plastic residues (Dong et al., 2024), and chemical fertilizers (Brown et al., 2021). However, studies focusing on the effects of CC on soil microbial metabolites and metabolic pathways are limited, necessitating further research (Cui et al., 2024).

*P. quinquefolius*, a medicinal plant valued for its economic and health benefits, is increasingly cultivated across China. Its cultivation follows a continuous 4-year cycle, but CC introduces severe challenges, such as soil-borne diseases, high seedling mortality, stunted plant growth, and reduced yield and quality. However, the microbial mechanisms driving CCOs in *P. quinquefolius* cultivation remain unclear. In this study, we aimed to investigate the effects of different CC durations on soil physicochemical properties, enzyme activities, microbial biomass and community structures, and metabolite profiles in the rhizosphere of *P. quinquefolius*. The main objectives were to (i) analyze the responses of soil bacterial communities, metabolite composition, and microbial activities across different years of *P. quinquefolius* CCOs, (ii) evaluate potential correlations between microbial community structures and changes in metabolites and metabolic functions, and (iii) elucidate the micro-mechanisms underlying CCOs in *P. quinquefolius*-planted soils. We hypothesized that CCOs significantly altered soil physicochemical properties and microbial community structures, leading to changes in metabolite profiles that contributed to the observed decline in soil health and increased prevalence of soil-borne diseases. Overall, this study applied UHPLC-MS/MS non-targeted metabolomics combined with Illumina high-throughput sequencing technologies to accurately assess the risks of CCOs in *P. quinquefolius* rhizosphere soil and provided the scientific basis for the management of crop cropping practices.

## 2 Materials and methods

### 2.1 Experimental design and soil sample collection

The experimental field with long-term CC of *P. quinquefolius* was located in Jixi Village (37°4’N, 122°59’E) in Wendeng County (Shandong, China). This region is characterized by a warm temperate monsoon climate, with an annual average temperature of 11.9 °C and annual precipitation of 767.8 mm. The soil was classified as fluvo aquic, which is a type of soil that is formed under conditions of fluctuating water tables and characterized by a well-developed profile with distinct horizons, good structure, and high fertility (Xin et al., 2019). The *P. quinquefolius* population was maintained at 500,000 ha^–1^, with 10 cm spacing between plants and 20 cm between rows. Due to serious CCOs, the farmland would be abandoned if the *P. quinquefolius* plants were cultivated for 4 years. To ensure the representativeness and comparability of the samples, four soil sample sites were selected from fields where *P. quinquefolius* had been continuously grown for 1 year (designated as CC1), 2 years (CC2), 3 years (CC3), and 4 years (CC4), respectively. In addition, a control site (CC0) with *Zea mays*-*Triticum aestivum* rotation cultivation was chosen at the same location to provide a baseline for comparison. The total area of both the *Zea mays*-*Triticum aestivum* rotation plot and the *P. quinquefolius* CC plot was 120 m × 120 m (14,400 m^2^). Within each plot, three subplots of 20 m × 20 m were selected for soil sampling, with five 1.0 m × 1.0 m quadrats randomly set up in each subplot for collecting rhizosphere soil samples. The soil samples from each quadrat were mixed to form composite samples, resulting in a total of 15 samples (five subplots × three biological replicates). The distance between each two soil sample sites was more than 500 m, and the soil samplings were collected in September 2023. Importantly, all experimental plots were previously under the same *Zea mays*-*Triticum aestivum* rotation system with uniform fertilization and management practices, ensuring consistent soil conditions at the start of the study. After passing through a 2-mm sieve, the soil samples were divided into three groups. The first group was air-dried in laboratory for 2 weeks to analysis the physicochemical properties including soil pH, cation exchange capacity (CEC), SOM, TN, NO_3_^–^, ammonium (NH_4_^+^), AP, and AK contents AP. The second group of fresh soils was used to count living bacteria, fungi, and Actinomyces; analyze soil enzyme activities; and measure microbial biomass carbon (MBC) and nitrogen (MBN). Immediately after collection, the samples were placed in sealed containers and stored in a –20 °C freezer for no longer than 4 weeks prior to analysis. This method of cryogenic preservation is according to the protocol described by Pavlovska et al. (2021). The third group was preserved at -80 °C for subsequent bacterial community and metabolic function analyses. All analyses were conducted in triplicates.

### 2.2 Quantification of soil microbial numbers

The soil bacteria, fungi, and Actinomyces in each sample were quantified using the plate counting method (Thornton et al., 1934). Briefly, tenfold serial dilutions were prepared by suspending 10 g of soil in 90 mL of sterile water, mixing thoroughly, and diluting to a final concentration of 10^–6^. Aliquots (0.1 mL) of these dilutions were spread onto plates containing potato-dextrose agar for fungi, beef extract peptone agar for bacteria, and Gauze’s No. 1 agar for Actinomyces. The plates were incubated at 37 °C for 24 h for fungi and at 25 °C for 48 h for bacteria and Actinomyces (Liao et al., 2018). Subsequently, the number of bacteria, fungi, and Actinomyces was counted.

### 2.3 Measurement of soil physicochemical properties

Soil pH was determined by measuring the pH of a 1:2.5 soil-to-water mixture using a glass electrode pH meter (PHS-25, Leici, Shanghai, China). Soil CEC was assessed using the silver thiourea method (Pleysier and Juo, 1980). The Walkley–Black method was used to quantify SOM (Walkey and Black, 1934), and an elemental analyzer was used to measure TN, NO_3_^–^-N, and NH_4_^+^ contents (CHNOS Elemental Analyzer, Vario EL, Elementar, Germany). The Olsen method was used to assess soil AP (Olsen and Sommers, 1982), and flame emission spectrometry was used to evaluate soil available potassium (AK) content (Jackson and Mahmood, 1992).

### 2.4 Analysis of soil enzyme activities

To assess soil health, we measured the activities of six key soil enzymes: urease (UREA), invertase (INV), alkaline phosphatase (ALP), catalase (CAT), protease (PRO), and polyphenol oxidase (PPO). In this experiment, UREA activity was quantified using a colorimetric assay for ammonium (NH_4_^+^) (Kandeler and Gerber, 1988). Briefly, 5.0 g of fresh soil was incubated with 10 mL of 10% urea solution and 1 mL of toluene at 37 °C for 24 h. After incubation, 1 mL of the supernatant was mixed with 4 mL of sodium phenate solution and 3 mL of sodium hypochlorite solution. The absorbance of the resulting blue color was measured at 578 nm using a spectrophotometer (UV-2600, Shimadzu, Japan). The INV activity was determined using the glucose colorimetric method (Gopal et al., 2007). A 5.0 g soil sample was incubated with 15 mL of 8% sucrose solution and 1 mL of toluene at 37 °C for 24 h. After incubation, 1 mL of the supernatant was mixed with 3 mL of 3,5-dinitrosalicylic acid reagent and heated in a boiling water bath for 5 min. The absorbance was measured at 540 nm. The ALP activity was assessed by monitoring the hydrolysis of p-nitrophenyl phosphate and the release of p-nitrophenol (Tabatabai and Bremner, 1969). A 1.0 g soil sample was incubated with 4 mL of 0.05 M p-nitrophenyl phosphate solution and 1 mL of toluene at 37 °C for 1 h. After incubation, 1 mL of 0.5 M CaCl_2_ and 4 mL of 0.5 M NaOH were added, and the mixture was filtered. The absorbance of the filtrate was measured at 400 nm. The CAT activity was measured using the potassium permanganate titration method (Trasar-Cepeda et al., 1999). Specifically, 2.0 g of soil was mixed with 40 mL of distilled water and 5 mL of 0.3% H_2_O_2_ solution, then incubated at 25 °C for 30 min. The reaction was stopped by adding 5 mL of 1.5 M H_2_SO_4_. The remaining H_2_O_2_ was titrated with 0.02 M KMnO_4_ solution. The activity of PRO was determined using the leucine aminopeptidase assay (Ladd and Butler, 1972). A 1.0 g soil sample was incubated with 5 mL of 1% casein solution and 1 mL of toluene at 37 °C for 2 h. After incubation, 2 mL of 0.5 M trichloroacetic acid was added to stop the reaction, and the mixture was filtered. The amino acid content in the filtrate was determined using the ninhydrin method, with absorbance measured at 570 nm. The PPO activity was measured using the orthobenzene-triol colorimetric method (Montgomery and Sgarbieri, 1975). For this, 1.0 g of soil was incubated with 5 mL of 0.1 M catechol solution and 1 mL of phosphate buffer (pH 6.0) at 30 °C for 2 h. The reaction was stopped by adding 2 mL of 0.5 M H_2_SO_4_. The absorbance of the supernatant was measured at 490 nm. Microbial biomass carbon (MBC) and nitrogen (MBN) were quantified using the chloroform fumigation-extraction method (Vance et al., 1987). Briefly, 20 g of fresh soil was divided into two subsamples. One subsample was fumigated with ethanol-free chloroform for 24 h at 25 °C in the dark, while the other served as a non-fumigated control. After fumigation, both fumigated and non-fumigated samples were extracted with 80 mL of 0.5 M K_2_SO_4_ solution by shaking for 30 min and then filtered. The organic carbon and total nitrogen in the extracts were determined using a total organic carbon analyzer (TOC-VCPH, Shimadzu, Japan) and a Kjeldahl nitrogen analyzer (Kjeltec 8400, FOSS, Sweden), respectively. MBC and MBN were calculated using the following equations:


$$M\,B\,C=\frac{\mathrm{Fumigated}C--\mathrm{Non}-\mathrm{fumigated}C}{K_{C}}$$

$$M\,B\,N=\frac{\mathrm{Fumigated}N--\mathrm{Non}-\mathrm{fumigated}N}{K_{N}}$$

where *K_C_* (0.45) and *K_N_* (0.54) are the conversion factors for carbon and nitrogen, respectively.

### 2.5 DNA extraction and sequencing

Genomic DNA was extracted from fresh soil (0.5 g) using a FastDNA SPIN Kit (Macherey-Nagel, Germany), according to the manufacturer’s protocol. DNA quality and purity were evaluated using a NanoDrop spectrophotometer (NanoDrop Technologies, Wilmington, DE, United States). The V4–V5 region of the bacterial 16S rRNA gene was amplified using the primers 515F (5′-GTGCCAGCMGCCGCGGTAA-3′) and 907R (5′-CCGTCAATTCMTTTRAGTTT-3′) (Bates et al., 2011; Parada et al., 2016). After PCR amplification and purification, paired-end sequencing was performed using the Illumina MiSeq platform. Raw sequencing data were processed using Quantitative Insights Into Microbial Ecology Version 2, employing the DADA2 pipeline to obtain amplicon sequence variants (ASVs). Taxonomic annotation of each ASV was performed using the SILVA database. The raw sequencing read data generated in this study have been deposited in the NCBI Sequence Read Archive (SRA) under the accession number SUB15654061.

### 2.6 Non-targeted metabolite analysis

Non-targeted metabolomic profiling was conducted using ultra-performance liquid chromatography-tandem mass spectrometry (UPLC-MS/MS). Thawed soil samples at 4 °C were mixed with a pre-chilled solvent mixture of methanol, acetonitrile, and water (2:2:1 volume ratio) and added to 100 mg of each sample. This mixture was vortexed, sonicated at low temperature for 30 min, exposed to -20 °C for 10 min, and centrifuged at 14,000 × g for 20 min at 4 °C. The supernatant was vacuum-dried, and the residue was re-dissolved in 100 μL of a 1:1 acetonitrile/water solution and then re-centrifuged at 14,000 × g for 15 min at 4 °C. The supernatant was used as the sample for the UPLC-MS/MS analysis. Quality control (QC) samples prepared by pooling 10 μL of each sample were processed and analyzed with the experimental samples to ensure consistency and reproducibility. The mass spectrometer was operated in both positive and negative electrospray ionization modes. Metabolites with > 50% missing values in any treatment group were excluded. Metabolite identification was achieved by matching the m/z values (within 10 ppm tolerance) and MS/MS spectral data with an in-house database of authenticated standards.

### 2.7 Data processing and statistical analysis

One-way analysis of variance (ANOVA) was performed using the SPSS 25 software (SPSS Inc., United States), followed by Tukey’s HSD *post-hoc* test to identify significant differences (*p* < 0.05) in soil physicochemical properties, enzymatic activities, and microbial biomass among various soil samples. Bacterial alpha diversity indices (ACE, Chao, Shannon, and Simpson) were calculated using the “vegan” package in R, with intergroup diversity differences assessed by the Kruskal–Wallis test. The bacterial community composition at the genus and phylum levels was visualized using Circos software.^1^ LEfSe analysis was performed to identify biomarker taxa in different soil samples using the “microbiomeMarker” package. Principal coordinates analysis (PCoA) and non-metric multidimensional scaling (NMDS) based on the Bray–Curtis distance and ANOSIM were used to elucidate differences in bacterial community composition. Redundancy analysis (RDA) was used to evaluate the correlations between the soil microbial community structure and soil microbial biomass, physicochemical properties, and enzymatic activities. The function of the bacterial community was predicted using FAPROTAX and the PICRUSt2 software. Bacterial co-occurrence networks based on relative ASV abundance were constructed using the Gephi software (version 10.1).

Statistical analyses of global metabolic variations among comparable groups were performed using Pareto-scaled principal component analysis (PCA) and orthogonal projections to latent structure discriminant analysis (OPLS-DA). Metabolites with significant differences (differential metabolites; DM) among groups were identified based on a variable importance in projection (VIP) score of > 1.0 and a *p*-value of < 0.05. DM was visualized using a heatmap generated using the “pheatmap” R package. The top 10 enriched metabolic pathways were illustrated using bubble plots from the KEGG database. Time-series analysis was performed using the “maSigPro” R package to identify metabolites with significant fluctuations across cultivation periods. Associations between soil bacterial communities and metabolite profiles were assessed using Procrustes analysis (via the “psych” package in R v4.2.2). Additionally, partial least squares-path modeling (PLS-SEM) performed using the “plspm” package was used to evaluate the direct and indirect effects of soil physicochemical properties and enzymatic activities on microbial community composition, metabolite profiles, and biomass.

## 3 Results

### 3.1 Effect of *P. quinquefolius* CCOs on soil physicochemical properties

*P. quinquefolius* CC significantly influenced the rhizospheric soil physicochemical properties (Table 1). Compared to those in the control (CC0), soil pH, CEC, SOM, TN, and NH_4_^+^ content in CC1, CC2, CC3, and CC4 treatments exhibited a progressive and significant decrease. Specifically, soil pH decreased by 12.2–28.0%, CEC by 42.6–65.5%, SOM by 8.7–27.3%, TN by 7.6–27.8%, and NH_4_^+^ by 16.9–56.6% with increasing years of continuous cropping. This consistent decline across multiple key indicators suggests a severe degradation of soil quality under prolonged monoculture. In contrast, the nitrate (NO_3_^–^) content showed a substantial increase, ranging from 36.0 to 75.6% across the CC treatments compared to CC0. This inverse relationship between NH_4_^+^ and NO_3_^–^ suggests altered nitrogen cycling dynamics, potentially due to inhibited nitrification or increased denitrification under CC stress. The contents of AP and AK initially increased, reaching their highest values in the CC2 treatment (52.3 and 152.4 mg kg^–1^, respectively), but then decreased with further prolonged cropping. These findings collectively highlight a significant and detrimental shift in the soil’s chemical environment, indicating reduced fertility and nutrient imbalance as a direct consequence of continuous *P. quinquefolius* cultivation. Influence of CCOs on soil enzyme activities.

**TABLE 1:** Changes of soil physicochemical properties in different continuous *Panax quinquefolius* L. cropping treatments.

Treatments	pH value	CEC (cmol g^–1^)	SOM (g kg^–1^)	TN (g kg^–1^)	NO_3_^–^-N (mg kg^–1^)	NH_4_^+^-N (mg kg^–1^)	AP (mg kg^–1^)	AK (mg kg^–1^)
CC0	6.29 ± 0.39 a	91.08 ± 0.8 a	16.1 ± 0.8 a	1.44 ± 0.16 a	17.2 ± 1.1 d	26.7 ± 1.8 a	28.1 ± 3.0 d	80.0 ± 1.7 d
CC1	5.52 ± 0.10 b	52.3 ± 0.6 b	14.7 ± 0.2 b	1.33 ± 0.10 ab	23.4 ± 1.7 c	22.2 ± 1.5 b	42.4 ± 2.1 c	91.2 ± 1.7 c
CC2	4.53 ± 0.03 c	48.4 ± 0.03 b	13.6 ± 0.2 bc	1.20 ± 0.08 bc	25.9 ± 1.7 b	21.3 ± 1.1 b	52.3 ± 2.7 a	152.4 ± 4.2 a
CC3	4.75 ± 0.03 c	39.6 ± 2.3 c	12.5 ± 1.0 cd	1.12 ± 0.13 bc	29.3 ± 0.1 a	14.7 ± 1.1 c	46.3 ± 1.5 b	149.6 ± 3.5 a
CC4	4.53 ± 0.07 c	31.4 ± 4.4 d	11.7 ± 0.9 d	1.04 ± 0.11 c	30.2 ± 1.0 a	11.6 ± 0.7 d	41.1 ± 0.04 c	117.6 ± 2.4 b

Values are the mean of three replicates. Different letters in the same column indicate that values are significantly different from each other at *p* ≤ 0.05. CEC, cation exchange capacity; SOM, soil organic matter; TN, total nitrogen; NO_3_^–^-N, nitrate-nitrogen; NH_4_^+^-N, ammonium-nitrogen; AP, available phosphorus; AK, available potassium.

### 3.2 Influence of *P. quinquefolius* CCOs on soil enzyme activities

The effects of *P. quinquefolius* CC on the activity of the six key soil enzymes are presented in Table 2. A consistent and progressive decrease in the activities of all measured enzymes-UREA, CAT, PPO, INV, PRO, and ALP-was observed with increasing years of *P. quinquefolius* cultivation. Compared to the CC0 treatment, the activities of UREA, CAT, PPO, INV, PRO, and ALP in CC1, CC2, CC3, and CC4 treatments decreased by 1.8–36.1%, 13.5–60.6%, 13.5–32.7%, 22.6–64.5%, 6.6–94.7%, and 35.3–89.4%, respectively. The most pronounced reductions were observed in PRO and ALP activities, particularly in the CC4 treatment, indicating a severe impairment of protein and phosphorus cycling. This widespread reduction in enzyme activities across all six indicators suggests a significant inhibition of overall soil microbial metabolic activity and a decline in the soil’s capacity to facilitate essential biogeochemical processes.

**TABLE 2:** Effects of continuous *Panax quinquefolius* L. cropping on soil enzymatic activities.

Treatments	UREA (μ g d^–1^ g^–1^)	CAT (μ g h^–1^ g^–1^)	PPO (nmol h^–1^ g^–1^)	INV (μ g d^–1^ g^–1^)	PRO (μ g h^–1^ g^–1^)	ALP (nmol h^–1^ g^–1^)
CC0	305.6 ± 6.9 a	60.9 ± 0.5 a	693.7 ± 12.9 a	6.2 ± 0.01 a	7.6 ± 0.3 a	244.3 ± 14.2 a
CC1	300.0 ± 5.2 a	52.7 ± 1.4 b	600.2 ± 10.2 b	4.8 ± 0.1 b	7.1 ± 0.6 ab	158.0 ± 3.0 b
CC2	244.8 ± 3.8 b	40.5 ± 2.2 c	527.5 ± 17.9 c	3.9 ± 0.0 c	6.6 ± 0.3 b	37.4 ± 3.1 c
CC3	215.4 ± 2.5 c	34.1 ± 1.8 d	529.4 ± 8.3 c	3.2 ± 0.1 d	3.7 ± 0.2 c	32.6 ± 1.9 c
CC4	195.3 ± 5.1 d	24.0 ± 2.0 e	467.2 ± 9.4 d	2.2 ± 0.1 e	0.4 ± 0.1 d	25.9 ± 1.7 c

Values are the mean of three replicates. Different letters in the same column indicate that values are significantly different from each other at *p* ≤ 0.05. UREA, urease activity; CAT, catalase activity; PPO, polyphenol oxidase activity; INV, invertase activity; PRO, protease activity; ALP, alkaline phosphatase activity.

### 3.3 Effect of *P. quinquefolius* CCOs on soil microbial numbers and biomass

As shown in Supplementary Figure 1, with increasing years of *P. quinquefolius* CC, the number of soil bacteria and fungi in the different sample soils continuously decreased. This decline suggests a negative impact of prolonged monoculture on the overall abundance of these crucial microbial groups. In contrast, the number of Actinomyces species showed a consistent increasing trend, indicating a potential shift in microbial dominance or a greater resilience of Actinomyces to CC stress. Furthermore, MBC and MBN showed no significant differences between the CC0 and CC1 treatments, suggesting that the initial year of continuous cropping had a limited impact on overall microbial biomass. However, MBC and MBN increased markedly in the CC2, CC3, and CC4 treatments (Supplementary Figure 2). Compared with those in the CC0 treatment, MBC and MBN increased by 19.1 and 35.4% in the CC2 treatment, 26.2 and 63.1% in the CC3 treatment, and 50.8 and 71.8% in the CC4 treatment, respectively. This unexpected increase in MBC and MBN, despite the decrease in bacterial and fungal numbers, could imply a shift toward microbial communities with higher biomass per cell or altered physiological states, possibly in response to accumulating root exudates or changes in nutrient availability.

### 3.4 Soil microbial community dynamics in response to CCOs

The effect of *P. quinquefolius* CC on soil microbial communities was evaluated by high-throughput 16S rRNA gene sequencing using the Illumina MiSeq platform. Analysis of 15 rhizospheric soil samples generated 862,744 high-quality sequences and identified 48,540 ASVs (Supplementary Table 1). The average number of ASVs in CC0, CC1, CC2, CC3, and CC4 treatments were 4,378; 4,525; 2,545; 2,439; and 2,285; respectively.

**FIGURE 1:**
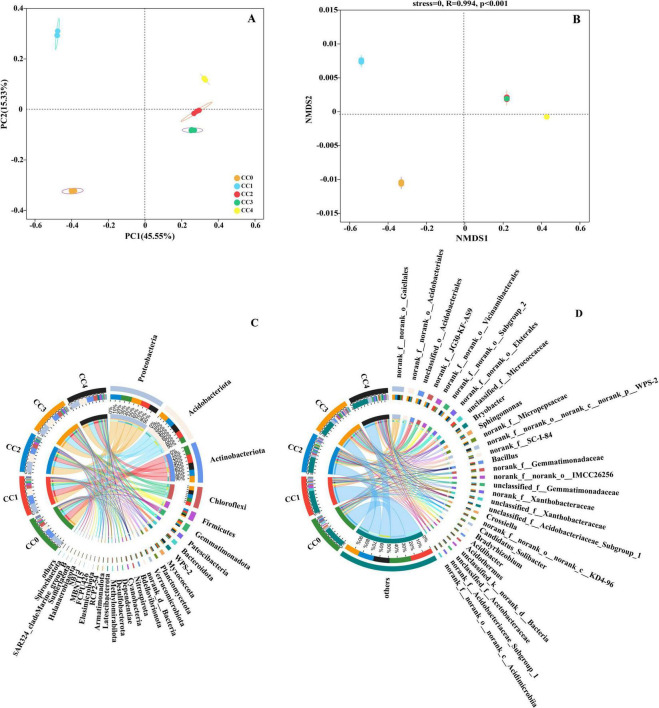
Principal coordinates analysis (PCoA) and non-metric multidimensional scaling (NMDS) of bacterial communities in *Panax quinquefolius* L. rhizosphere soil. **(A)** PCoA analysis and **(B)** NMDS analysis illustrating the clustering of bacterial communities at the amplicon sequence variant (ASV) level, based on Bray-Curtis dissimilarity. **(C)** Circle diagram showing the relative abundance of dominant bacterial phyla and (**D**) dominant bacterial genera across different continuous cropping treatments (CC0, CC1, CC2, CC3, CC4). CC0 represents the control (maize-wheat rotation), while CC1-CC4 represent continuous cropping for 1–4 years, respectively.

The effects of *P. quinquefolius* CC on ACE, Chao, Shannon, and Simpson indices representing the microbial community richness and diversity are shown in Table 3. The Ace and Chao indices showed no significant differences between CC0 and CC1 but were significantly decreased in CC2, CC3, and CC4 treatments. The Shannon index remained unaffected in the CC1 and CC2 treatments but significantly increased in the CC3 and CC4 treatments. Conversely, the Simpson index exhibited a remarkable decrease in the CC4 treatment group. The analyses of PCoA and NMDS demonstrated distinct clustering of the 15 soil samples into five groups, with bacterial communities in CC2, CC3, and CC4 separated from those in CC1 and CC0 (Figures 1A, B). Circos graphs illustrate the bacterial compositions of both phyla (Figure 1C) and genus levels (Figure 1D). The dominant bacterial phyla included Proteobacteria (24.5–29.6%), Acidobacteriota (14.8–24.4%), Actinobacteriota (16.9–24.5%), Chloroflexi (9.6–11.3%), and Firmicutes (3.1–6.1%). At the genus level, the most abundant taxa were *Gaiellales*, *Acidobacteriales*, *JG30-KF-AS9*, and *Vicinamibacterales*.

**TABLE 3:** The richness and diversity of microbial community in in different continuous *Panax quinquefolius L.* cropping soils.

Treatments	Alpha diversity indexes			
	Ace	Chao	Shannon	Simpson
CC0	34.8 ± 3.3 a	34.7 ± 3.2 a	2.06 ± 0.06 b	0.186 ± 0.017 a
CC1	36.2 ± 2.1 a	36.0 ± 2.0 a	2.03 ± 0.04 b	0.170 ± 0.003 ab
CC2	28.7 ± 1.1 b	28.3 ± 1.2 b	2.04 ± 0.02 b	0.185 ± 0.009 a
CC3	26.3 ± 0.6 b	26.3 ± 0.6 b	2.14 ± 0.02 a	0.182 ± 0.001 ab
CC4	22.9 ± 0.2 c	22.3 ± 1.2 c	2.13 ± 0.03 a	0.163 ± 0.006 b

Values are the mean of three replicates. Different letters in the same column indicate that values are significantly different from each other at *p* ≤ 0.05. Ace, Abundance-based Coverage Estimator; Chao, Chao1 richness estimator; Shannon, Shannon-Wiener diversity index; Simpson, Simpson’s diversity index.

**FIGURE 2:**
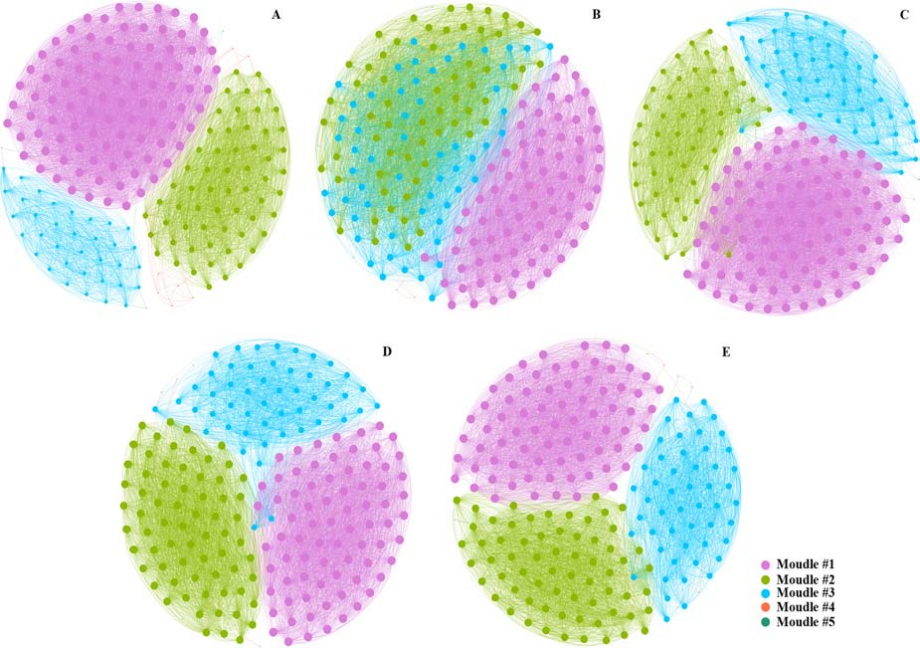
Bacterial co-occurrence networks in *Panax quinquefolius* L. rhizosphere soils under continuous cropping. Networks for **(A)** control (CC0), **(B)** 1 year (CC1), **(C)** 2 years (CC2), **(D)** 3 years (CC3), and **(E)** 4 years (CC4) of continuous cropping. Each node represents a unique amplicon sequence variant (ASV). The size of each node is scaled by its degree (number of connections). Nodes sharing the same color belong to the same module. Edges between nodes signify strong correlations (Spearman’s rank correlation coefficient, *r* > 0.08, *P* < 0.05).

Co-occurrence networks of soil bacterial communities were constructed at the ASV level to analyze bacterial co-occurrence patterns across various CC treatments of *P. quinquefolius* (Figure 2). The network revealed five distinct ecological phylotype clusters. Compared with CC0, with increased duration of CC, significant reductions were observed in network metrics: the number of edges decreased from 6,760 to 6,065; the average weighted degree declined from 67.9 to 61.6, and the network density decreased from 0.343 to 0.314. By contrast, the modularity index increased from 0.506 to 0.663. Furthermore, the ratio of positive edges decreased in the CC1, CC2, CC3, and CC4 treatments by 0.5, 1.9, 4.3, and 2.6%, respectively, compared to that in CC0 (Supplementary Table 2). These results suggest that *P. quinquefolius* CC significantly disrupts network complexity and destabilizes network stability.

**FIGURE 3:**
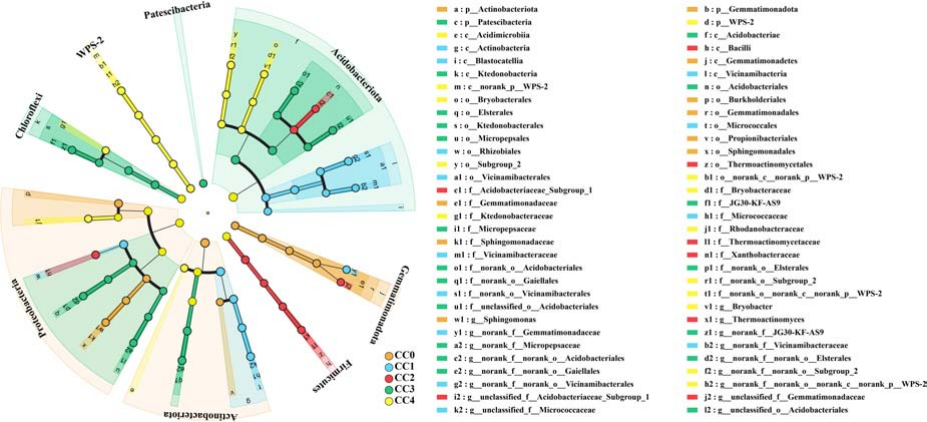
Linear discriminant analysis effect size (LEfSe) identifying bacterial biomarkers in *Panax quinquefolius* L. rhizosphere soil across continuous cropping years. The taxa with significantly different relative abundances among the CC0, CC1, CC2, CC3, and CC4 treatments are represented by colored dots (*p* < 0.05). The dots from the center to the outside represents phylum, class, order, family and genus, which have an effect size LDA score > 4.

Pairwise comparisons among the five treatments using LEfSe with a threshold of LDA > 4 identified 64 biomarkers spanning from the phylum to the genus level (Supplementary Figure 3). The findings indicated that CC treatments significantly reduced the relative abundance of Gemmatimonadota, Actinobacteria, and Proteobacteria phyla compared to that in the control treatment (Figure 3). In the CC1 treatment group, Actinobacteria, Blastocatellia, and Vicinamibacteria were abundant at the class level. The CC2 treatment resulted in the enrichment of Firmicutes and Thermoactinomycetales. Acidobacteriota, Chloroflexi, Acidobacteriae, and Ktedonobacteria were dominant in the CC3 treatment group. In the CC4 treatment, the relative abundance of WPS-2, Bryobacterales, Ktedonobacteraceae, Bryobacteracea, and Rhodanobacteraceae was significantly increased.

**FIGURE 4:**
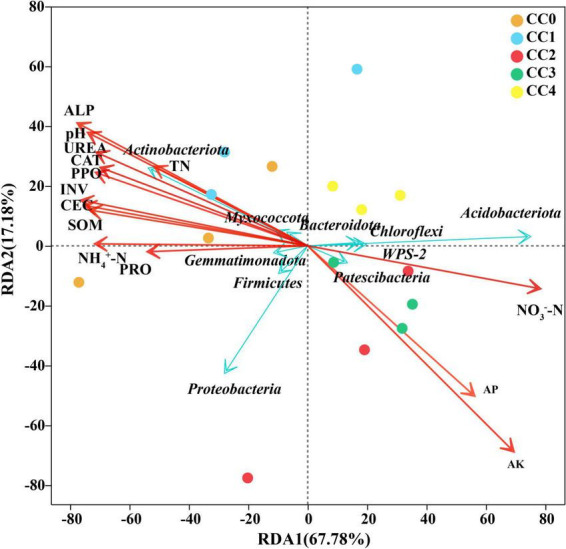
Redundancy analysis (RDA) biplot showing the relationships among soil physicochemical properties, enzymatic activities, and the top 10 most abundant bacterial phyla. Arrows represent environmental variables (physicochemical properties and enzyme activities), with their length indicating the strength of correlation and direction indicating the gradient. Points represent bacterial phyla. The angle between an environmental variable arrow and a phylum indicates the correlation between them.

Evaluation of the relationship between the relative abundances of the top 10 dominant bacterial phyla and various soil environmental variables using RDA revealed that soil environmental variables, including physicochemical characteristics and enzyme activities, accounted for 85.0% of the variation observed in the bacterial community (Figure 4). On the left side of the RDA1 axis, Acidobacteriota, Chloroflexi, Patescibacteria, and WPS-2 were positively correlated with AP, AK, and NO_3_^–^-N. Conversely, on the right side of the RDA1 axis, Actinobacteriota, Myxococcota, Bacteroidota, and Gemmatimonadota were positively correlated with pH, CEC, SOM, TN, NO_4_^+^-N, UREA, ALP, CAT, INV, PRO, and PPO. Among all soil environmental factors, AK (*R*^2^ = 0.89, *p* = 0.001), pH (*R*^2^ = 0.64, *p* = 0.004), and ALP (*R*^2^ = 0.72, *p* = 0.003) were identified as the primary factors influencing the structure of the soil bacterial community (Supplementary Table 3).

**FIGURE 5:**
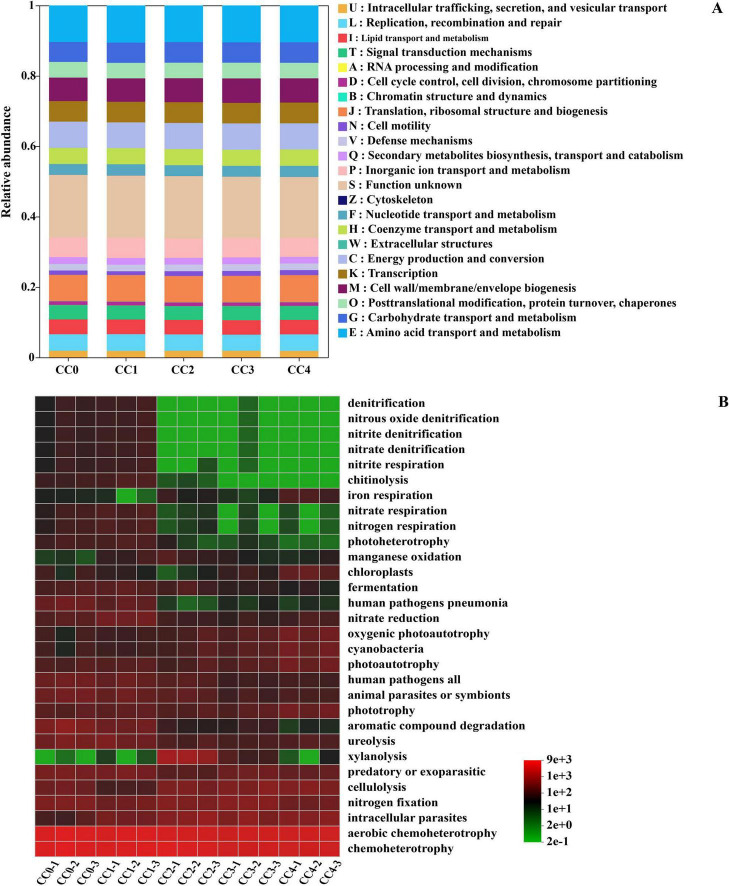
Predicted metabolic functions of the bacterial community in *Panax quinquefolius* L. rhizosphere soil under continuous cropping. **(A)** Functional prediction based on PICRUSt2 analysis, showing enriched metabolic pathways. **(B)** Functional prediction based on FAPROTAX analysis, illustrating specific biogeochemical functions. Both analyses compare the bacterial communities across different continuous cropping treatments.

Mapping the predicted functional profiles to the cluster of orthologous groups (COGs) database using the PICRUSt2 functional prediction tool indicated that the microbial functions across all treatments were primarily enriched in categories such as amino acid transport and metabolism, translation, ribosomal structure and biogenesis, inorganic ion transport and metabolism, and energy production and conversion (Figure 5A). Among these, amino acid transport and metabolism exhibited the highest relative abundance, comprising 10.38% in CC0, 10.54% in CC1, 10.41% in CC2, 10.47% in CC3, and 10.45% in CC4.

Further elucidation of the metabolic capabilities of the microbial communities was achieved by referencing the FAPROTAX database, which identified 30 distinct functional groups within the study period (Figure 5B). Bacteria exhibiting chemoheterotrophy and aerobic chemoheterotrophy constituted a substantial proportion of the soil microbial community with no significant differences among the treatments. Conversely, the nitrogen cycling functions, specifically denitrification, nitrous oxide denitrification, nitrite denitrification, nitrate denitrification, nitrate respiration, and nitrogen respiration, differed significantly among the CC2, CC3, CC4, CC0, and CC1 treatments.

### 3.5 Response of microbe non-targeted metabolomic profiling to CCOs

Metabolomic analysis identified 1,040 distinct compounds, including 635 positively and 405 negatively charged metabolites. These compounds were predominantly classified into eicosanoids, fatty acids, amino acids, nucleosides, and phospholipids (Supplementary Figure 4). The results of PCA and OPLS-DA indicated a remarkable differentiation between the control and treatment groups (Supplementary Figure 5), suggesting that *P. quinquefolius* CC duration significantly altered the soil metabolite profile. This alteration, in turn, negatively affected the metabolic pathways and overall functioning of the soil microbial community. Furthermore, the CC2, CC3, and CC4 groups clustered closely together but remained distinct from the CC0 and CC1 groups.

**FIGURE 6:**
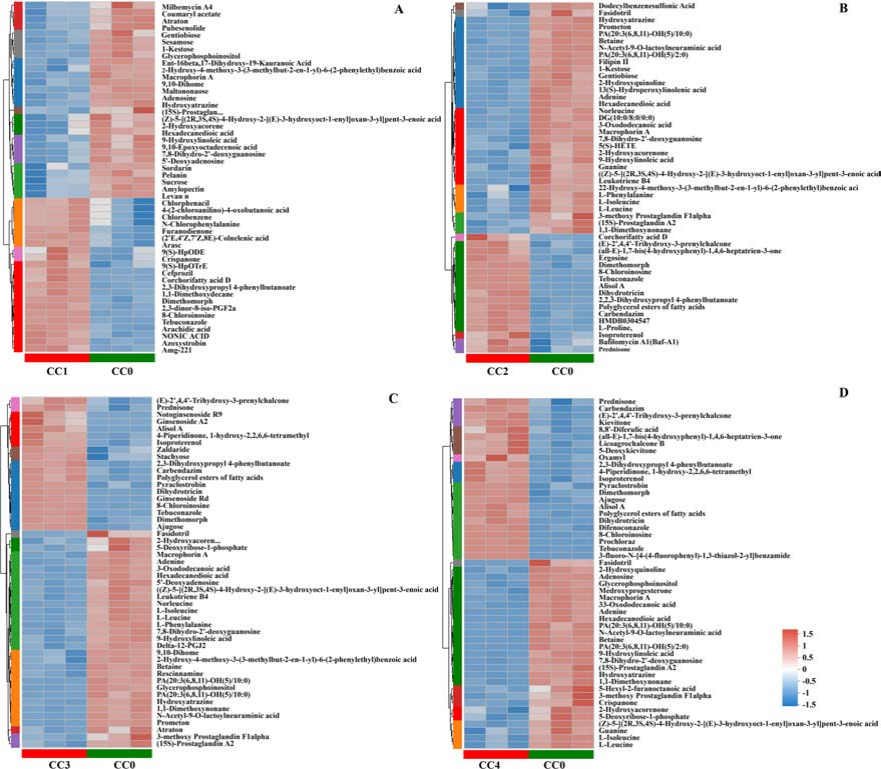
Cluster heatmaps of differentially abundant metabolites (DMs) in *Panax quinquefolius* L. rhizosphere soil. Heatmaps illustrate the relative abundance of DMs when comparing **(A)** 1 year continuous cropping (CC1) vs. control (CC0), **(B)** 2 years continuous cropping (CC2) vs. control (CC0), **(C)** 3 years continuous cropping (CC3) vs. control (CC0), and **(D)** 4 years continuous cropping (CC4) vs. control (CC0).

The metabolite profile differences between the treatment and control groups were further analyzed to identify the specific metabolic changes responsible for the observed separation. The PLS-DA score plot showed a clear distinction in both positive and negative metabolites for the CC1 vs. CC0, CC2 vs. CC0, CC3 vs. CC0, and CC4 vs. CC0 groups (Supplementary Figure 6). Using the multivariate OPLS-DA model (VIP > 1 and *p* < 0.05), we identified 289 (123 upregulated, 159 downregulated) DMs in CC1 vs. CC0, 364 (140 upregulated, 224 downregulated) in CC2 vs. CC0, 374 (129 upregulated, 245 downregulated) in CC3 vs. CC0, and 386 (143 upregulated, 243 downregulated) in CC4 vs. CC0 (Supplementary Figure 7). These DMs were classified into eight categories: peptides, steroids, carbohydrates, hormones and transmitters, lipids, nucleic acids, organic acids, and vitamins and cofactors (Supplementary Figure 8). A Venn diagram comparing metabolic sets (Supplementary Figure 9) identified 80 unique DMs in CC1 vs. CC0, 33 in CC2 vs. CC0, 27 in CC3 vs. CC0, 34 in CC4 vs. CC0, and 137 common DMs across all the groups. The top 50 DMs in each group are illustrated in cluster heatmaps (Figure 6).

**FIGURE 7:**
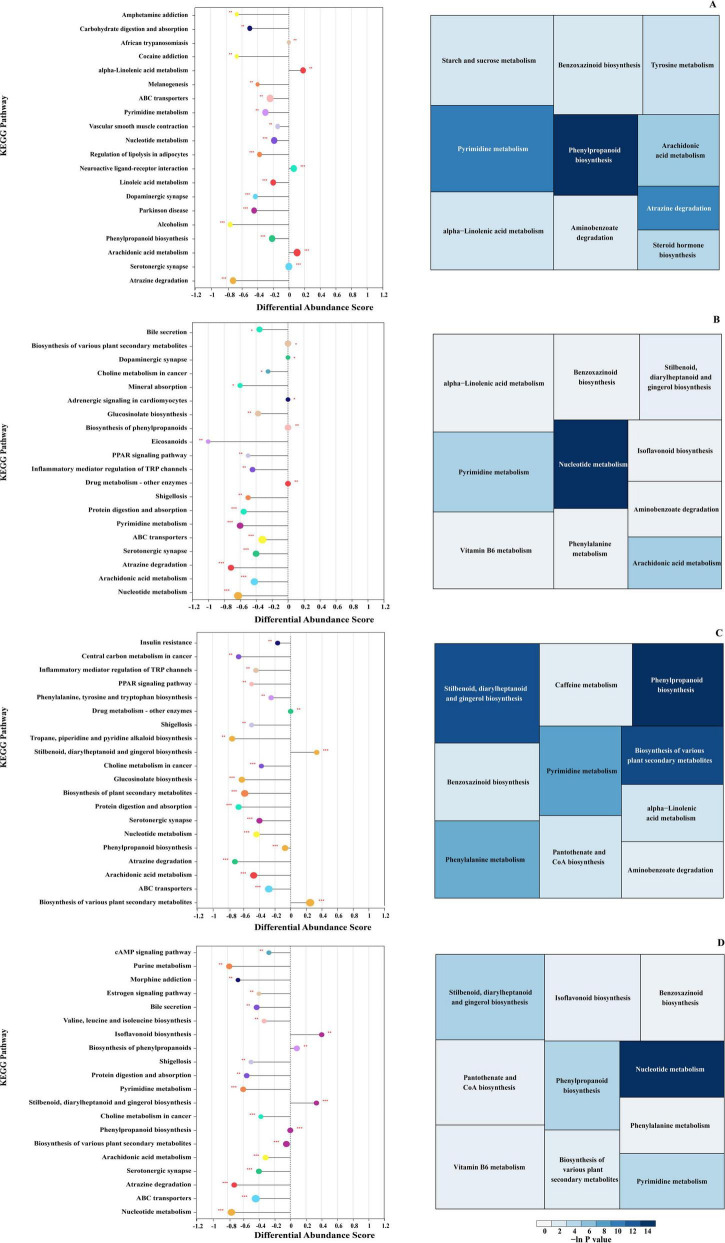
KEGG enrichment analysis of metabolic pathways for differentially abundant metabolites (DMs) in *Panax quinquefolius* L. rhizosphere soil. Enriched pathways are shown for comparisons of **(A)** 1 year continuous cropping (CC1) vs. control (CC0), **(B)** 2 years continuous cropping (CC2) vs. control (CC0), **(C)** 3 years continuous cropping (CC3) vs. control (CC0), and **(D)** 4 years continuous cropping (CC4) vs. control (CC0). The size of each box represents the pathway’s influence degree, and the color indicates the enrichment degree. Statistical significance is denoted by asterisks: *p < 0.05, **p < 0.01, ***p < 0.001.

The confirmed DMs were mapped to the KEGG database to identify their corresponding metabolic pathways. Twenty metabolic pathways with *p*-values of < 0.05 in each group are shown in Figure 7. The calculated differential abundance score (DA score) was significantly downregulated in most pathways across the four groups (DA score < 0). In the CC1 vs. CC0 groups, DMs were enriched in phenylpropanoid biosynthesis, pyrimidine metabolism, atrazine degradation, and arachidonic acid metabolism (Figure 7A). For CC2 vs. CC0 groups, pyrimidine, arachidonic acid, alpha-linolenic acid, and nucleotide metabolisms were enriched (Figure 7B). The CC3 vs. CC0 group showed enrichment in pyrimidine metabolism; phenylpropanoid biosynthesis; various plant secondary metabolite biosynthesis; stilbenoid, diarylheptanoid, and gingerol biosynthesis; and phenylalanine metabolism (Figure 7C). In the CC4 vs. CC0 group, the primary metabolic pathways were nucleotide, stilbenoid, diarylheptanoid and gingerol, phenylpropanoid, and pyrimidine metabolism (Figure 7D).

**FIGURE 8:**
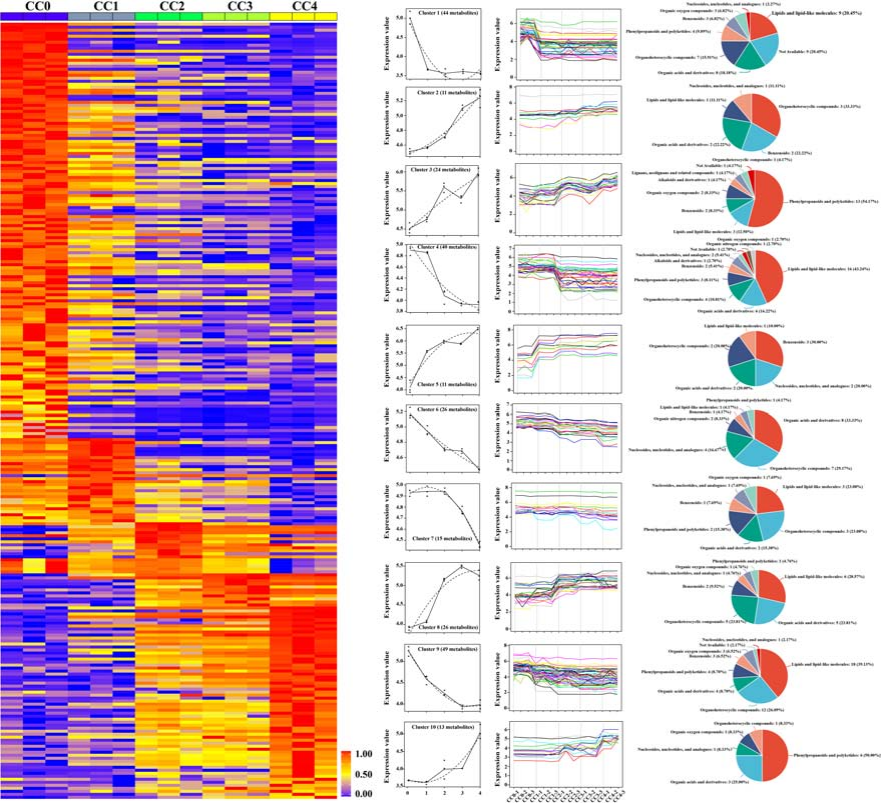
Time-series expression patterns of differential metabolites in *Panax quinquefolius* L. rhizosphere soil across continuous cropping years. This heatmap, generated by maSigPro chronological analysis, illustrates the dynamic changes in metabolite abundance over 0, 1, 2, 3, and 4 years of continuous cropping. Metabolites are grouped into ten distinct clusters based on their expression patterns. The accompanying pie charts show the classification of identified metabolites within each cluster according to the Human Metabolome Database (HMDB).

A time series analysis using maSigPro was conducted to identify metabolite changes across the five periods of *P. quinquefolius* CC. The heatmap (Figure 8) revealed significant stereoselectivity of metabolites among the different cropping years. Ten significant gene clusters were also identified. The metabolites in Clusters 2, 3, 5, 8, and 10 increased with prolonged cropping, whereas those in Clusters 1, 4, 6, 7, and 9 decreased. According to the HMDB classification, upregulated metabolites included organoheterocyclic compounds, benzenoids, and organic acids and derivatives in Cluster 2; phenylpropanoids, polyketides, lipids, and lipid-like molecules in Cluster 3; benzenoids, nucleosides, nucleotides, analogs, and organic acids and derivatives in Cluster 4; lipids, organic acids, and organoheterocyclic compounds in Cluster 8; and phenylpropanoids and polyketides in Cluster 10. Downregulated metabolites were primarily lipids, organic acids, and organoheterocyclic compounds in Cluster 1; lipids and organic acids in Cluster 4; organic acids and organoheterocyclic compounds in Cluster 6; and lipids and organoheterocyclic compounds in Clusters 7 and 9.

### 3.6 Association between the microbes and metabolites in the rhizosphere soil of *P. quinquefolius*

**FIGURE 9:**
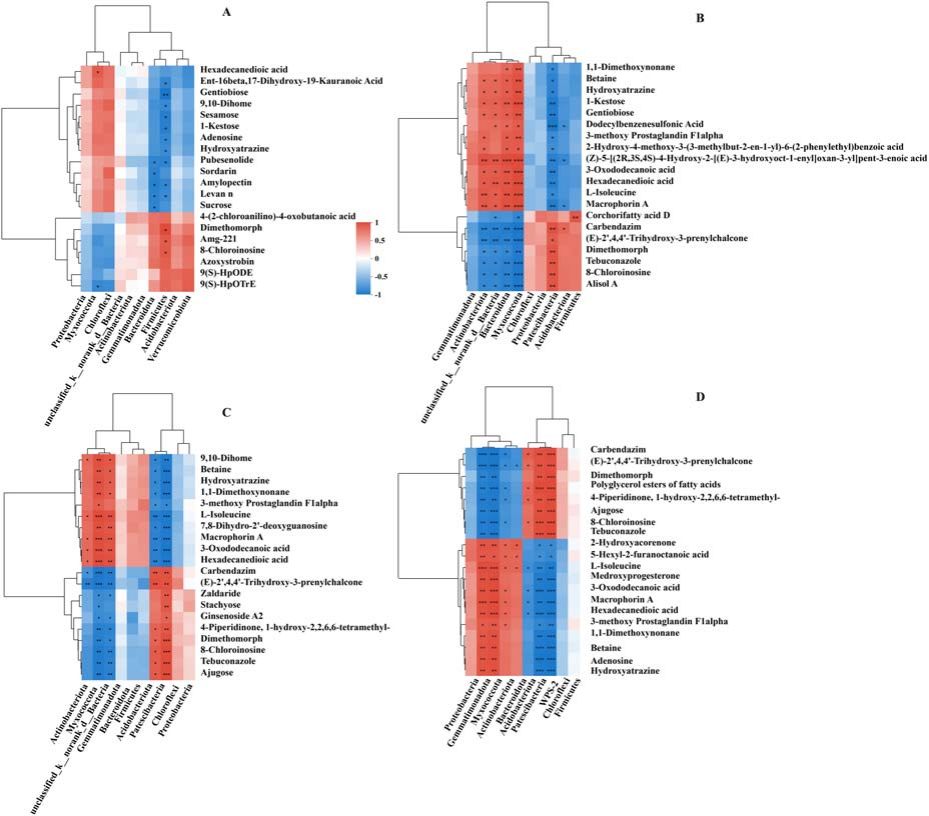
Pearson correlation analysis between dominant soil bacterial phyla and altered soil metabolites in *Panax quinquefolius* L. rhizosphere soil. Correlation matrices are presented for comparisons of **(A)** 1 year continuous cropping (CC1) vs. control (CC0), **(B)** 2 years continuous cropping (CC2) vs. control (CC0), **(C)** 3 years continuous cropping (CC3) vs. control (CC0), and **(D)** 4 years continuous cropping (CC4) vs. control (CC0). Color intensity indicates the strength and direction of correlation (e.g., red for positive, blue for negative). Statistical significance is denoted by asterisks: *p < 0.05, **p < 0.01, ***p < 0.001.

Procrustes analysis was conducted to identify the potential relationships between soil bacteria and metabolites (Supplementary Figure 10). The analysis revealed no significant relationship between the CC1 and CC0 groups (Supplementary Figure 10A, M2 = 0.713, *p* > 0.05); however, significant correlations were found between CC2 and CC0 (Supplementary Figure 10B, M2 = 0.393, *p* < 0.05), CC3 vs. CC0 (Supplementary Figure 10C, M2 = 0.248, *p* < 0.05), and CC4 vs. CC0 groups (Supplementary Figure 10D, M2 = 0.291, *p* < 0.05). Pearson’s correlation analysis further examined the relationships within the paired microbiome-metabolome datasets (Figure 9). In the CC1 vs. CC0 group, Bacteroidota was negatively correlated with pubesenolide, sordarin, amylopectin, levan n, and sucrose. *Firmicutes* showed a significant positive correlation with dimethomorph, amg-221, and 8-chloroinosine but a negative correlation with ent-16beta,17-dihydroxy-19-kauranoic acid, gentiobiose, 9,10-dihome, sesamose, 1-ketose, adenosine, hydroxyatrazine, pubesenolide, amylopectin, and levan n. In the CC2 vs. CC0 group, Actinobacteriota, Bacteroidota, Myxococcota, and Patescibacteria were highly correlated with most metabolites (except corchorifatty acid D). In the CC3 vs. CC0 group, Myxococcota was significantly negatively correlated with 9,10-dihome, betaine, hydroxyatrazine, 1,1-dimethoxynonane, 3-methoxy prostaglandin F1α, L-isoleucine, 7,8-dihydro-2’-deoxyguanosine, macrophorin A, 3-oxododecanoic acid, and hexadecanedioic acid but positively correlated with other metabolites. Acidobacteriota and Patescibacteria showed opposite correlations compared to Myxococcota. In the CC4 vs. CC0 group, all the selected metabolites were highly correlated with Gemmatimonadota, Myxococcota, Patescibacteria, and WPS-2.

### 3.7 Correlations between soil metabolites, microbes, and environmental factors

**FIGURE 10:**
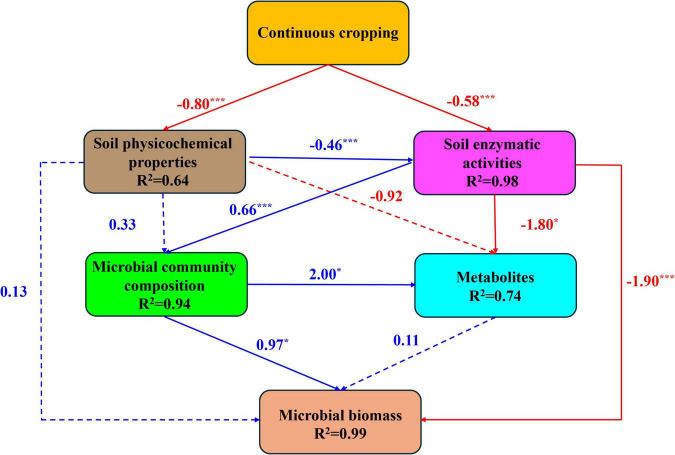
Partial least squares path modeling (PLS-SEM) illustrating the effects of continuous *Panax quinquefolius* L. cropping on soil physicochemical properties, enzymatic activities, bacterial communities, metabolite profiles, and microbial biomass. Red lines indicate a positive correlation (promoting effect), while blue lines indicate a negative correlation (inhibition effect). The numbers adjacent to the lines represent the standardized path coefficients. Statistical significance is denoted by asterisks: *p < 0.05, ***p < 0.001.

Mantel’s test demonstrated the relationships between the bacterial community, diversity, metabolite profiles, soil physiochemical properties, enzyme activities, and microbial biomass (Supplementary Figure 11). Soil environmental factors were significantly correlated with the bacterial community composition, diversity, and metabolite profiles, with UREA, ALP, and MBN notably influencing the bacterial community. Metabolites were strongly associated with UREA, ALP, pH, and AK but showed a weaker correlation with PRO and TN. Bacterial diversity was significantly related to all environmental factors except for AP. The analysis of PLS-SEM revealed the effect of *P. quinquefolius* CC on the rhizosphere microbial community (Figure 10). The goodness-of-fit index of the model was 0.82, suggesting a well-constructed influence pathway. *P. quinquefolius* CC negatively affected soil physicochemical properties (path coefficient = -0.80, *p* < 0.001) and soil enzymatic activities (path coefficient = 0.46, *p* < 0.001). Changes in soil physicochemical properties did not directly affect microbial community composition, metabolites, or microbial biomass, whereas soil enzymatic activity positively influenced microbial community composition and negatively affected metabolites and microbial biomass. Additionally, microbial community composition positively affected microbial biomass and metabolites, whereas changes in metabolites did not directly influence microbial biomass (path coefficient = 0.11, *p* > 0.05).

## 4 Discussion

### 4.1 Detrimental effects of CCOs on soil physicochemical properties and enzyme activities

Continuous cultivation of the same crop significantly alters soil physicochemical properties, including pH and nutrient levels, thereby affecting subsequent crop health and growth (Zhao et al., 2024). Studies have revealed a notable decrease in pH in soils continuously cultivated with *Camellia sinensis* (Arafat et al., 2020), *Nicotiana tabacum* (She et al., 2017), and *Cucumis melo* (Li J. et al., 2023). Tan et al. (2021) demonstrated that *Nicotiana tabacum* CC for 10 years depleted soil nutrients such as SOM, AP, TN, and NO_3_^–^-N. Similarly, *Zea mays* CCOs reduced trace elements such as manganese and iron in *Zea mays* systems (Jiao et al., 2019). Consistent with these studies, our study also revealed significant decreases in soil pH, SOM, CEC, TN, and NH_4_^+^-N contents with prolonged *P. quinquefolius* cultivation. Soil acidification in CC systems results from excessive chemical N fertilizers, accumulation of root exudates, such as propionic, palmitic, and palmitoleic acids, and bio-decomposition of plant residues (Wang F. et al., 2022). Furthermore, CC depletes soil nutrients through plant uptake and inhibits C and N mineralization (Gentry et al., 2013), impairing nutrient transformation and cycling. This reduces the nutrient chelation capacity of the soil, consequently increasing nutrient loss via leaching and runoff (Hussain et al., 2019). However, AP and AK levels were higher in CCOs treatments than those in the control. In agreement with this, Gu et al. (2020) showed an increase in total phosphorus, total potassium, AP, and AK with continuous Chinese chive cropping years, likely due to the high application of chemical P and K fertilizers, reduced soil CEC, and inhibited plant cation absorption capacity.

Soil enzymes are vital for nutrient cycling and energy transformation and serve as bioindicators for biochemical processes (Gong et al., 2019; Wyszkowska et al., 2019). CCOs have been shown to reduce soil enzyme activity, which indicates a decline in soil quality (Gong et al., 2019; Qin et al., 2017). This study observed a decrease in the levels of UREA, CAT, PPO, INV, PRO, and ALP with increasing years of *P. quinquefolius* CCOs, suggesting significant inhibition of soil microbial metabolic activity by CCOs. Soil CAT, crucial for microbial metabolism and detoxification of reactive oxygen species, showed reduced activity in continuously cropped soil, indicating restrained aerobic organism activity and inhibited scavenging of toxic substances (Cai et al., 2019). Soil UREA and PRO are key enzymes in the soil N cycle that catalyze urea decomposition and protein conversion into oligopeptides and amino acids (Greenfield et al., 2021). Soil ALP influences the catabolic transformation and bioavailability of organic P (Vives-Peris et al., 2020), whereas INV hydrolyzes sucrose to glucose and fructose, providing essential energy for microbial metabolism (Cheng et al., 2021). Soil PPO is involved in recalcitrant SOC decomposition (Sinsabaugh et al., 2005), and both enzymes participate in the soil C cycle (Abad-Valle et al., 2016). The reduction in UREA, PRO, ALP, INV, and PPO activity indicates that CCOs restrained the catabolic transformation and biological efficiency of N, P, and C metabolism in soil microecosystems, leading to reduced soil fertility for plant growth. Furthermore, CC-induced challenges suppress the expression of functional genes associated with key enzyme activities (Zhao et al., 2021), reduce the number of enzyme-producing microorganisms (Li J. et al., 2023), and alter microbial diversity and abundance (Yang et al., 2018), thereby resulting in low enzyme activity in long-term CC soil systems. We observed a strong positive correlation between soil enzyme activity and pH during *P. quinquefolius* CC. These results indicate that the decrease in soil pH due to CCOs may further contribute to the reduction in soil enzyme activity in continuously cropped soils (Liu et al., 2021).

### 4.2 Disruption of soil microbial community structure caused by CCOs

Soil microorganisms are essential for biogeochemical cycling, organic matter turnover, nutrient availability, and the promotion of plant growth (Mohanram and Kumar, 2019). The abundance, diversity, metabolic activity, and community structure of rhizosphere microorganisms indicate the soil health (Dong et al., 2016). Studies have shown that long-term CC negatively affects soil microbial populations, diversity, and richness compared with short-term CC practices (Li J. et al., 2023). Under CC, rhizospheric microbial populations showed a decreased diversity of beneficial bacteria and fungi and an increased diversity of pathogenic bacteria (Zhang H. et al., 2022). Gao et al. (2021) found a significant decrease in the total bacterial population in sweet potato fields with longer monoculture duration. Our study showed that *P. quinquefolius* CC significantly reduced bacterial and fungal numbers while increasing those of Actinomycetes, potentially affecting ecosystem function stability. Previous studies have highlighted the importance of soil microbial diversity in determining soil quality (Lehmann et al., 2020). An elevated Shannon index indicates increased microbial diversity, whereas a high Simpson index indicates reduced diversity (Zheng et al., 2018). Higher Chao1 and Ace indices indicate increased bacterial richness (Guo et al., 2021). In this study, the Ace, Chao, and Simpson indices significantly decreased, whereas the Shannon index showed an upward trend with increasing CC duration. These results indicated that *P. quinquefolius* CC reduced bacterial richness but increased bacterial diversity in the rhizosphere soil. Taxonomic classification of 16S rRNA gene sequences identified Proteobacteria, Acidobacteriota, Actinobacteriota, Chloroflexi, and Firmicutes as the dominant bacterial phyla in these soils. *Proteobacteria*, the most abundant phylum in soil (Militon et al., 2010), is essential for the global cycling of C, N, and S (Bezza and Chirwa, 2017). This phylum also degrades organic pollutants, suppresses plant diseases, and promotes plant growth (Feng et al., 2015). As Proteobacteria consists of nutrient-rich, fast-proliferating bacteria in soils high in organic C and N, their reduced presence in CC soils, due to decreased SOM and TN, hampers nutrient and energy cycling, weakening plant resilience to soil-borne diseases. Actinobacteria, which are associated with disease suppression, are more abundant in disease-suppressive soils than in disease-conducive soils (Mendes et al., 2011). The reduced abundance of Actinobacteria confirmed the presence of pathogenic microorganisms in CC samples. Acidobacteriota, an anaerobic phototrophic group, thrives in nutrient-poor environments (Ward et al., 2009) and is highly sensitive to nitrogen availability (Fierer et al., 2011). Their growth is inhibited in nitrogen-rich legume soils (Che et al., 2022). The inverse relationship between TN and NH_4_^+^-N contents and Acidobacteriota abundance corroborates this conclusion and may explain the increased Acidobacteriota abundance under nutrient-deficient conditions in *P. quinquefolius* CC. Chloroflexi, a phylum of oligotrophic bacteria with high stress tolerance, is prevalent in harsh nutrient-poor arid soils (Huang et al., 2019). An increase in Chloroflexi abundance typically signifies reduced soil quality, suggesting a decline in soil nutrients in *P. quinquefolius* CC. This finding aligns with that in previous studies on tomatoes (Jiang et al., 2024), *P. notoginseng* (Wang F. et al., 2022), and cut chrysanthemum (Li J. et al., 2023) under long-term monoculture, where CCOs increased the relative abundance of Chloroflexi. Similar to Proteobacteria, Firmicutes significantly suppresses soil-borne diseases, such as wilt (Lee et al., 2020). In this study, the relative abundance of Firmicutes increased during the first 2 years of *P. quinquefolius* CC and then declined in the third and fourth years, highlighting their role in pathogen antagonism during the initial cropping period. LEfSe analysis identified significant bacterial taxa linked to soil bacterial community changes over different years in *P. quinquefolius* CC, with distinct taxa in each group. The key microbial biomarkers shifted from Gemmatimonadota, Actinobacteriota, and Proteobacteria, thriving in favorable soil conditions, to Acidobacteriota, Chloroflexi, and WPS-2, which endure extreme environments, indicating soil quality deterioration over successive cropping years. Our study demonstrated that *P. quinquefolius* CC decreased the relative abundance of beneficial bacterial taxa such as Gaiellales, Vicinamibacteria, Sphingobacteriales, and Sphingomonas while increasing the abundance of pathogenic bacteria such as Acidobacteriales and Xanthomonadaceae.

Soil microorganism composition and dynamics are influenced by changes in soil environmental factors (Tan et al., 2021). Studies have demonstrated that bacterial populations thrive in high pH environments, whereas bacterial diversity decreases significantly under acidic conditions (Kim et al., 2020). Degrune et al. (2015) indicated that a soil pH variation of < 0.5 units can affect bacterial growth. Ma et al. (2022) also reported that continuous *Lilium brownii* cropping reduced rhizosphere microbial diversity and soil pH value. Furthermore, SOM and nutrients such as AP, TN, and NH_4_^+^-N significantly affect bacterial community variation in continuous *Nicotiana tabacum* cropping soils (Tan et al., 2021). In this study, RDA results showed that soil bacterial community structure was mainly influenced by pH, AK, and ALP activity, suggesting that soil acidification, K levels, and organic P transformation are crucial factors limiting microbial diversity and composition in *P. quinquefolius* CC soils. Additionally, pH was positively correlated with the abundance of Gemmatimonadetes and Actinobacteria and negatively correlated with that of Acidobacteriota, consistent with the findings of previous studies (Conradie and Jacobs, 2021; Ma et al., 2023).

Identifying symbiotic patterns in microbial communities is vital for understanding the potential relationships between microorganisms in terrestrial ecosystems (Banerjee et al., 2019). Co-occurrence network analysis is widely used to examine microbial community stability under external disturbances such as soil contamination (Song et al., 2024), chemical fertilizer application (Zhang H. et al., 2022), and CCOs (Zhang H. et al., 2022). In this study, co-occurrence network analysis revealed that CC treatments significantly reduced edges, average weighted degree, and network density while increasing the modularity index. These findings indicate that compared to non-CC, CCOs reduce network complexity and stability, resulting in a fragmented bacterial community (Li J. et al., 2023). Because complex soil microbial communities generally exhibit greater resistance and resilience to environmental changes than less diverse ones (Allison and Martiny, 2008), continuous *P. quinquefolius* cultivation makes the soil bacterial community more sensitive and difficult to restore when facing soil environmental changes. Additionally, with prolonged CC, the proportion of positive correlations among bacterial interactions in the rhizosphere soil network decreased from 54.5 to 50.2%, indicating increased competitive interactions among bacterial assemblages within the CC system.

Prediction of the effects of *P. quinquefolius* CC on microbial biogeochemical functions revealed that amino acid transport and metabolism exhibited the highest relative abundances across all treatments and also demonstrated an increasing trend with increased CC duration. Similar findings have been observed in continuous *Sesamum indicum* cropping (Lyu et al., 2023) and *P. notogingseng* (Wang F. et al., 2022), where CCOs s led to an increase in amino acid transport and metabolism. Amino acids are crucial metabolites driving microbial metabolism and biosynthesis (Lin et al., 2017). Under adverse conditions, microbial communities can sustain high cell growth by reprogramming essential amino acid metabolism (Yang et al., 2021). The upregulation of amino acid transport and metabolism highlights the role of amino acids in the regulation of microbial metabolic activity, offering insights into enhancing microbial resistance to CCOs in consecutive *P. notogingseng* monocultures. The analyze of FAPROTAX effectively predicts microbial community functions in N, S, P, and C biotransformations under stress (Sansupa et al., 2021). In our study, the FAPROTAX functional prediction indicated that functional genes related to N cycling, including denitrification, nitrate, and nitrogen respiration, were significantly affected by the CC treatment. Soil N cycling disturbances were mainly due to changes in bacterial taxa, such as the order Burkholderiales, family Nitrosomonadacease, and class Acidimicrobiia, which are associated with N cycling and beneficial plant growth (Garrido-Oter et al., 2018). The decline in the relative abundance of soil bacteria due to *P. quinquefolius* CC suppressed nitrogen fixation and conversion, which is insufficient for plant growth. The phylum Nitrospirae and class Nitrospiria are crucial for the ammonia- and nitrite-oxidizing processes. The reduction of Nitrospirae and Nitrospiria indicates dissimilatory NO_3_^–^ reduction to NH_4_^+^ and inhibited denitrification (Jakus et al., 2021). These findings may explain the increased NO_3_^–^-N content and decreased NH_4_^+^-N levels in the *P. quinquefolius* CC treatments.

### 4.3 Metabolomic changes induced by CCOs

Soil metabolites originate from plant root exudates, microbial metabolites, and organic matter decomposition (Cheng et al., 2018), and changes in their composition and content reflect microbial responses to environmental stress (Wu et al., 2022). In the present study, metabolomics analysis identified 289 DMs in the CC1 vs. CC0 group, 364 DMs in the CC2 vs. CC0 group, 374 DMs in the CC3 vs. CC0 group, and 386 DMs in the CC4 vs. CC0 group. The identified metabolites were categorized as carbohydrates, nucleic acids, steroids, hormones and transmitters, peptides, vitamins, and cofactors, indicating responses to CCOs. Mapping of these DMs into the KEGG database revealed significant enrichment in phenylpropanoid biosynthesis (map00940) and pyrimidine metabolism (map00240) pathways across groups, consistent with previous studies (Shen et al., 2024). The phenylpropanoid pathway is crucial for the biosynthesis of compounds that protect cells from oxidative damage under abiotic stress, such as phenols, flavonoids, lignin, coumarin, hydroxycinnamic acid, cinnamic acid, ferulic acid, coumaroylshikimic acid, and caffeoylshikimic acid (Neelam et al., 2020; Sharma et al., 2019). The pyrimidine metabolism pathway is vital for microbial energy metabolism, such as the electron transport chain and TCA cycle (Xue et al., 2023), which not only participates in cell energy supply, DNA synthesis, and cell division and proliferation (Hu et al., 2022) but also plays a crucial role in improving cellular adaptation mechanisms in response to external stresses (Li J. et al., 2023). The downregulation of phenylpropanoid biosynthesis and pyrimidine metabolism in *P. quinquefolius* CC treatments suggests that the stress resistance of microbes and plants to CCOs was weakened due to the inhibited biosynthesis of phenolic compounds and shortened energy supply. Furthermore, the significant downregulation of various pathways, including starch and sucrose metabolism, tyrosine metabolism, isoflavonoid biosynthesis, nucleotide metabolism, and biosynthesis of various plant secondary metabolites, indicated that *P. quinquefolius* CC extensively affected primary and secondary metabolism in plant defense against pathogens (Lyu et al., 2023; Shen et al., 2024).

Time-series analysis revealed the temporal dynamics of soil metabolites in different CC treatments, demonstrating distinct variations among the five CC treatments. Of the 259 identified metabolites, 174 were downregulated and 85 were upregulated with increased CC duration. The downregulated metabolites were primarily lipids and lipid-like molecules, whereas the upregulated metabolites comprised organic acids and derivatives, phenylpropanoids, and polyketides. Lipids and lipid-like molecules, which are vital for cellular biofilms, membrane transport (Bramkamp and Lopez, 2015), and cellular signaling (Sunshine and Iruela-Arispe, 2017), are crucial for microbial resistance to environmental stress by maintaining membrane fluidity and homeostasis (Stankeviciute et al., 2019). The reduction in these metabolites suggests that CCOs impair the stress resistance of soil microorganisms. Accumulation of organic acids fosters pathogenic fungi and inhibits plant growth-promoting rhizobacteria (Wu et al., 2017), supported by the negative correlation between organic acids (e.g., betaine, 3-oxododecanoic acid, and L-isoleucine) and the abundance of Patescibacteria, WPS-2, and Acidobacteriota phyla. Furthermore, organic acid accumulation may lower soil pH by releasing H^+^ ions (Zhao et al., 2024). Phenylpropanoids and polyketides are key plant metabolites that detoxify harmful substances and enhance abiotic stress tolerance (Frémont et al., 2022). The increased levels of phenylpropanoids and polyketides (e.g., glicophenone, xanthomicrol, and demethoxycurcumin) suggest a photoprotective mechanism against CC-induced challenges in *P. quinquefolius*.

### 4.4 Microbe-metabolite interactions and the contribution to CCOs

As key drivers of soil metabolic activity, soil microorganisms influence metabolite stereoselectivity (Song et al., 2020). Exploring the relationship between differential metabolites and rhizosphere microbes is therefore crucial. Co-occurrence patterns between bacterial communities and metabolic profiles were examined in soils with varying CC durations. Procrustes analysis revealed an M2 value of 0.713 (*p* = 0.291) for CC1 vs. CC0 groups, indicating no significant correlation between bacterial communities and metabolic profiles during the first year of *P. quinquefolius* growth. As continuous cultivation increased, M2 decreased to 0.393 (*p* = 0.045) in the CC2 vs. CC0 group, 0.248 (*p* = 0.021) in the CC3 vs. CC0 group, and 0.291 (*p* = 0.038) in the CC4 vs. CC0 group, confirming a significant correlation between metabolism and bacterial communities after 2–4 years of cultivation. This correlation was strengthened with prolonged *P. quinquefolius* cropping. This finding aligns with those of previous studies on continuous soybean cropping (Cui et al., 2024), long-term *Camellia oleifera* cultivation (Lu et al., 2022), and heatmap correlation analysis. The positive association between certain metabolites may be attributed to their presence in microbial secretions, whereas the negative correlation between microbial taxa and metabolites can be explained by the specific metabolic consumption of these metabolites by microbes (Li H. et al., 2019).

The composition and dynamics of soil microorganisms and metabolites are significantly influenced by changes in soil environmental factors; however, their potential relationships remain unclear. The analysis of PLS-SEM was used to model the direct and indirect relationships between CCOs, soil physicochemical properties, enzymatic activities, microbial community composition, metabolites, and microbial biomass. The structural equation model showed that *P. quinquefolius* CC significantly and directly affected the soil physicochemical properties and enzymatic activities. These enzymatic activities significantly and positively influenced the microbial community and negatively affected soil metabolite profiles. Conversely, soil physicochemical properties showed no obvious relationship with the metabolite profiles of microbial community composition. These findings suggest that soil enzymatic activities, in conjunction with soil physicochemical properties, play a crucial role in regulating soil microorganisms, metabolite composition, and dynamics. Similarly, Zhao et al. (2017) concluded that the degradation of soil enzyme activity due to continuous cropping is a primary cause of *P. notoginseng* replanting failure. Microbial biomass was positively influenced by the microbial community and negatively influenced by soil enzymatic activities, suggesting that *P. quinquefolius* CC significantly altered the soil bacterial community composition, primarily because of enzymatic activity constraints, leading to disturbances in soil microbial biomass accumulation.

### 4.5 Limitations and considerations

It is important to note that the comparison between the *P. quinquefolius* monoculture soils (CC1–CC4) and the maize-wheat rotation soil (CC0) introduces a potential confounding variable: crop species. The observed differences in soil properties, microbial communities, and metabolite profiles between CC0 and the CC treatments may not be solely attributable to the CC practice. Instead, these differences could be partly due to the fundamental differences between the rhizosphere environments created by *Zea mays/Triticum aestivum* and *P. quinquefolius*. *Zea mays* and *Triticum aestivum*, as cereal crops, have different root exudates, nutrient demands, and associated microbial communities compared to *P. quinquefolius*, a medicinal plant. Therefore, the observed changes in soil characteristics and microbial dynamics could be influenced by both the CC practice and the specific crop species involved. To more accurately isolate the effects of CCOs on soil health and microbial ecology, future studies should consider using a more comparable control, such as a rotation system involving *P. quinquefolius* with other crops (e.g., legumes or other medicinal plants known to alleviate CCOs) (Cui et al., 2024; Zeeshan Ul Haq et al., 2023), or a fallow control within the same field type. Additionally, while our study provides a comprehensive snapshot of the rhizosphere at specific time points, the dynamic nature of soil microbial communities and metabolite profiles suggests that more frequent, longitudinal sampling could offer deeper insights into the temporal progression of CCOs (Dong et al., 2016). Future research could also benefit from integrating metagenomic or metatranscriptomic approaches to provide a more detailed functional understanding of the microbial community’s response to continuous cropping stress (Pang et al., 2021; Wang et al., 2025).

## 5 Conclusion

This study elucidates the microecological mechanisms underlying CCOs in *P. quinquefolius*, demonstrating a consequential decline in soil health marked by deteriorated physicochemical properties, dysbiosis of the microbial community, and pronounced alterations in metabolite profiles. Specifically, CCOs resulted in reduced soil pH, CEC, SOM, TN, and enzymatic activities, while concurrently promoting the proliferation of pathogenic taxa and disrupting beneficial microbial interactions. The inhibitory effects of CCOs were particularly evident in key microbial functions, such as impairing amino acid metabolism and nitrogen cycling processes, thus providing new mechanistic insights into strategies for enhancing microbial resilience. Metabolomic profiling further revealed significant disruptions in phenylpropanoid biosynthesis and pyrimidine metabolism, reflecting shifted microbial metabolic activities under continuous cropping stress. By integrating non-targeted metabolomics with high-throughput sequencing, this work offers a comprehensive perspective on soil ecosystem dysfunction and deliver novel insights for developing sustainable management practices aimed at mitigating CCOs and preserving soil health. Future research should prioritize long-term strategies to improve soil fertility and suppress soil-borne diseases in *P. quinquefolius* cropping systems.

## Data Availability

The raw data supporting the conclusions of this article will be made available by the authors, without undue reservation.

## Footnotes

1
http://circos.ca/
