# Supplementary material for: Effects of continuous cropping on soil metabolomics and rhizosphere bacterial communities in Panax quinquefolius L
Source: Front Microbiol. 2025 Nov 26;16:1698779. doi: 10.3389/fmicb.2025.1698779 (PMC12689531; doi:10.3389/fmicb.2025.1698779)
Supplement: Supplementary file 1 [file Data_Sheet_1.docx]

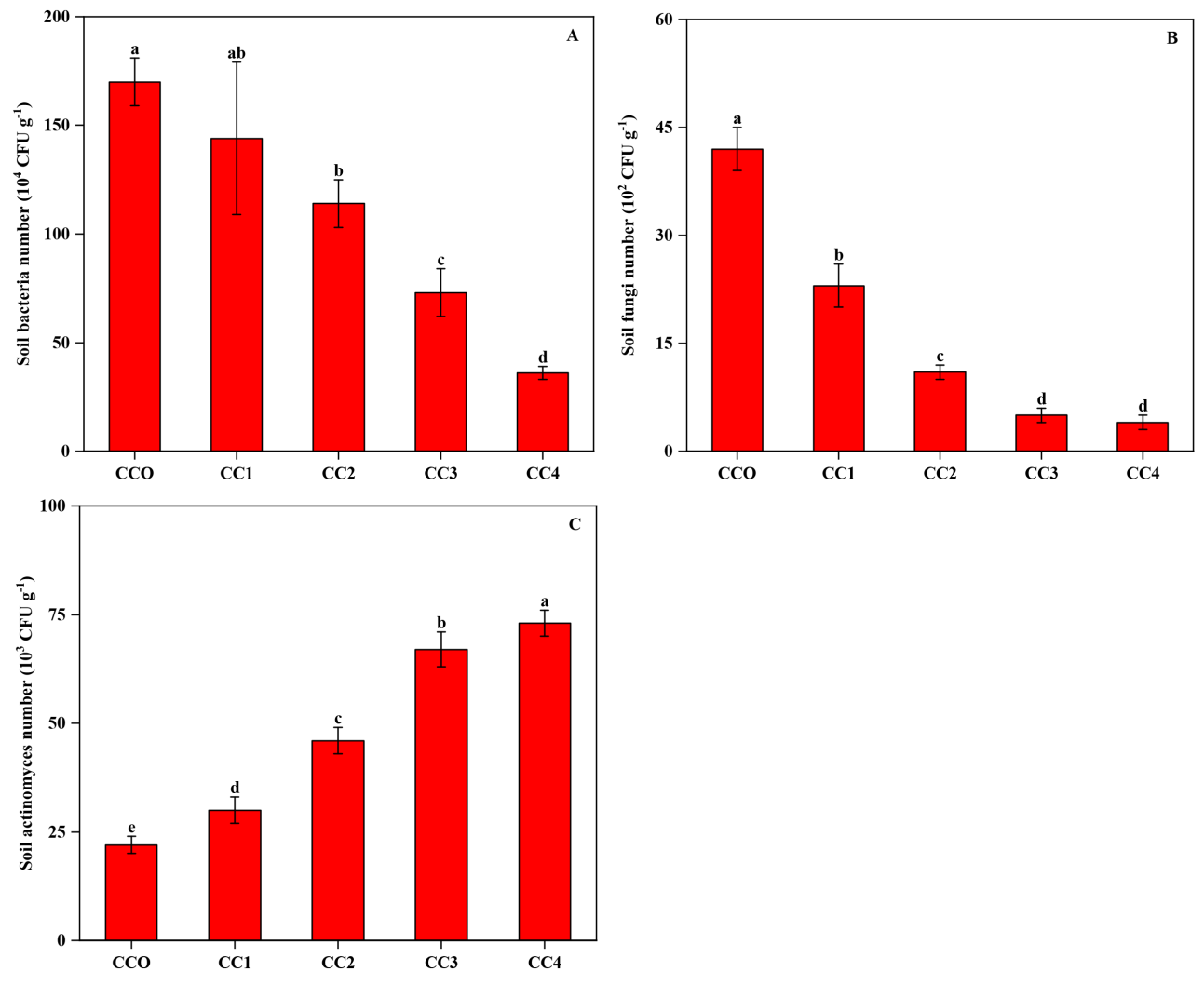


**Fig. S1 Quantification of soil microbial numbers in *Panax quinquefolius* L. rhizosphere soil under continuous cropping.** The number of (A) bacteria, (B) fungi, and (C) actinomyces in different continuous cropping treatments. Vertical bars represent the mean ± standard deviation (SD) of three replicates (n=3). Different letters above the bars indicate significant differences among treatments at *p* < 0.05.


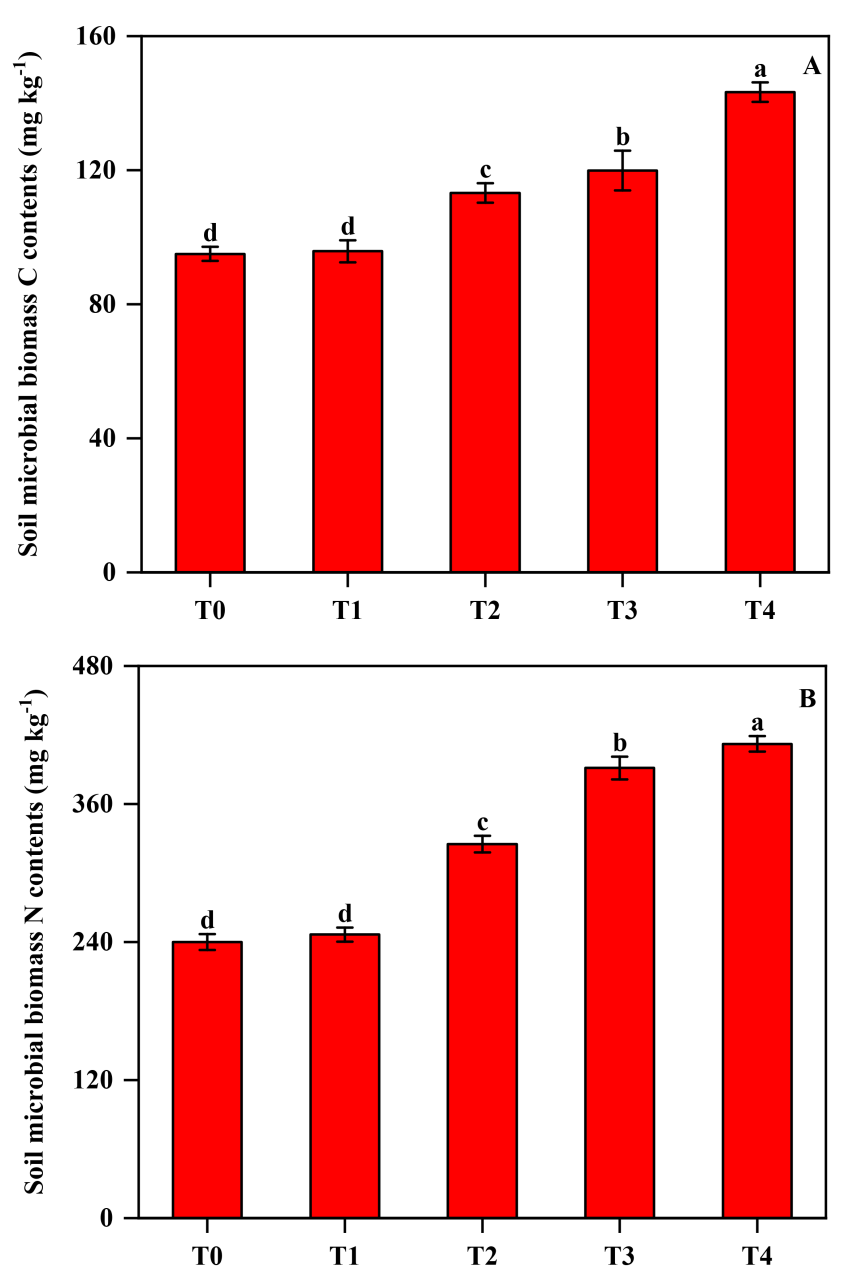


**Fig. S2 Effects of continuous *Panax quinquefolius* L. cropping on microbial biomass carbon (MBC) and microbial biomass nitrogen (MBN).** (A) MBC and (B) MBN in *Panax quinquefolius* L. rhizosphere soil under different continuous cropping treatments. Vertical bars represent the mean ± standard deviation (SD) of three replicates (n=3). Different letters above the bars indicate significant differences among treatments at *p* < 0.05."


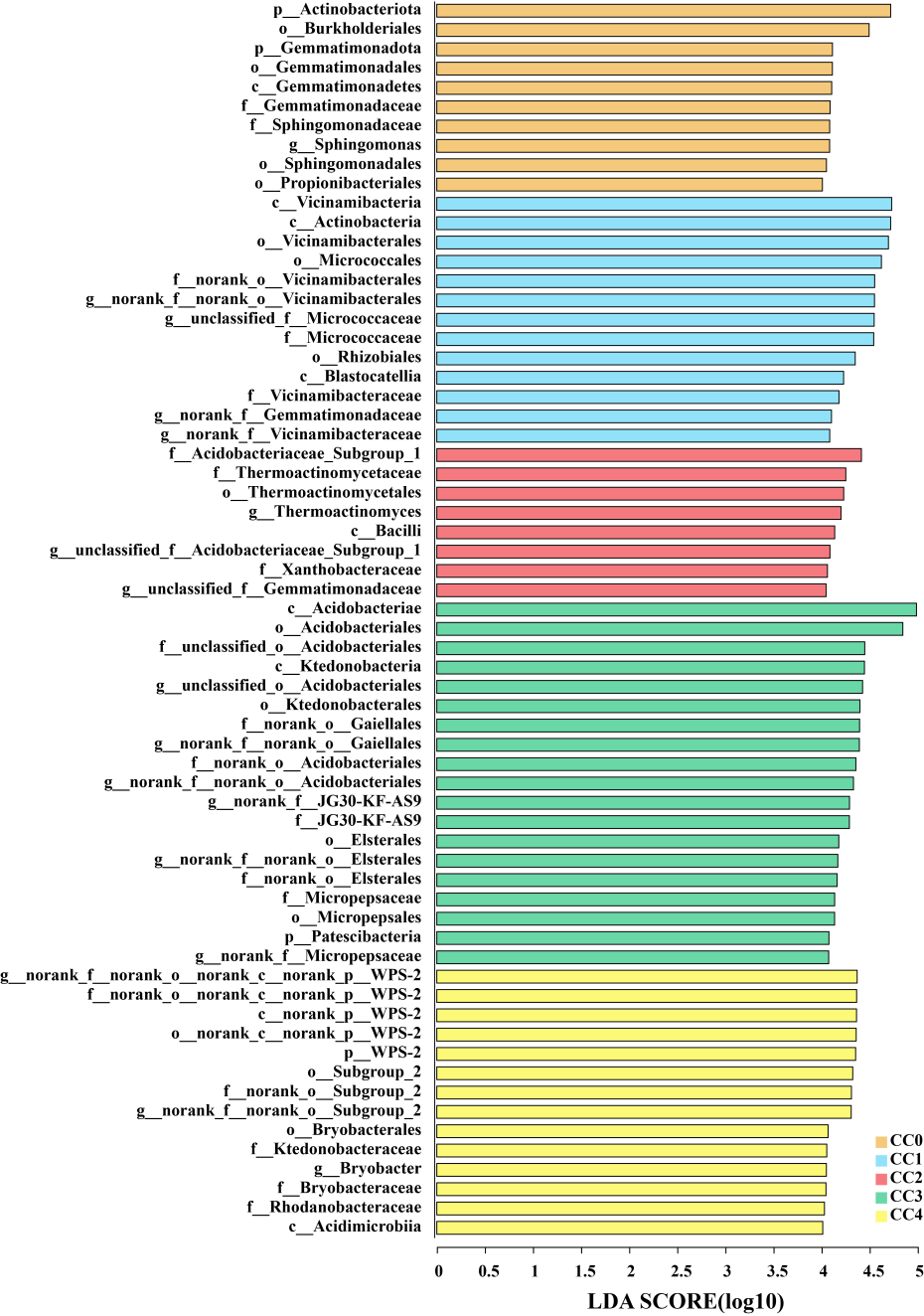


**Fig. S3 Indicator bacterial taxa identified by LEfSe analysis with LDA scores greater than 4.** These taxa show significantly different relative abundances in bacterial communities associated with *Panax quinquefolius* L. rhizosphere soil from different continuous cropping years.


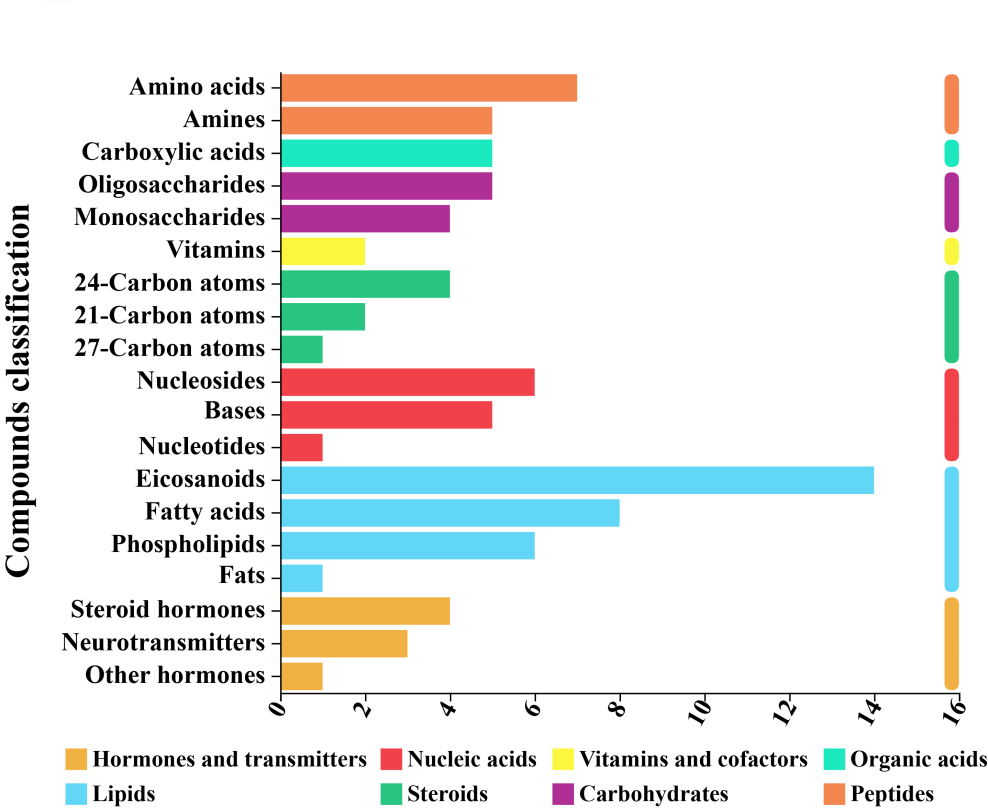


**Fig. S4 Compound classification of identified metabolites based on the Kyoto Encyclopedia of Genes and Genomes (KEGG) database.**

**
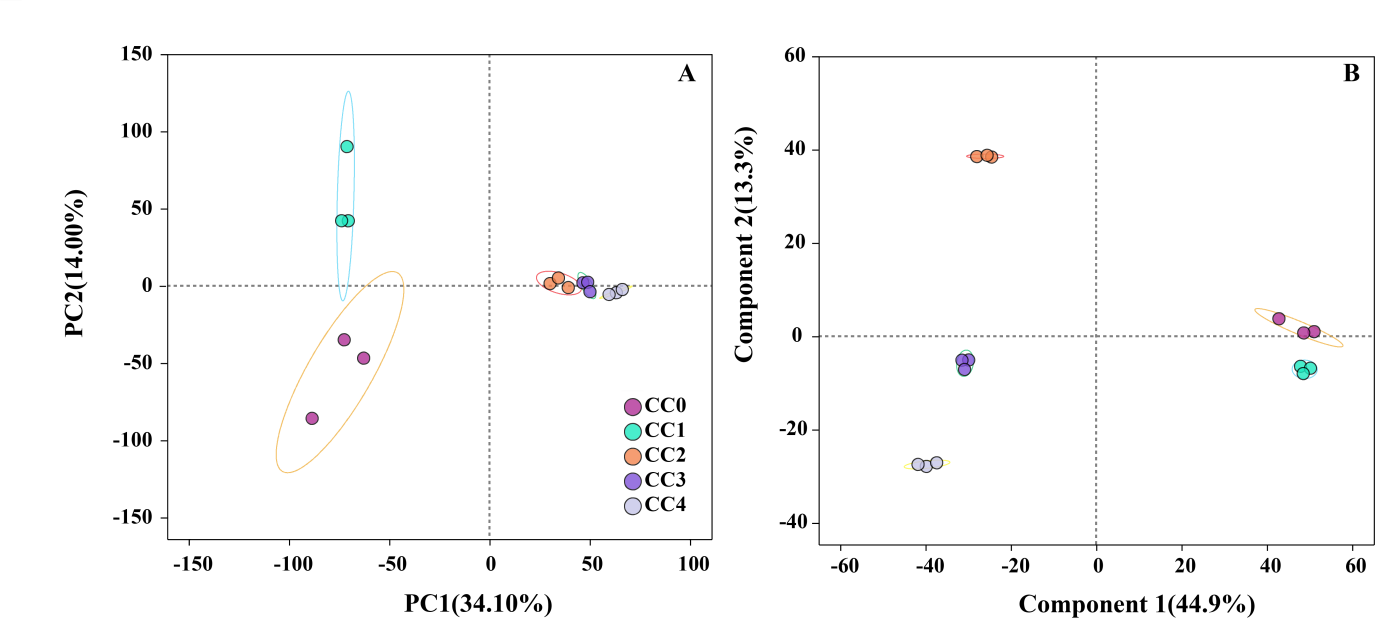
**

**Fig. S5 Principal Component Analysis (PCA) and Orthogonal Partial Least Squares Discriminant Analysis (OPLS-DA) of soil metabolites.** (A) PCA ordination analysis and (B) OPLS-DA scores plot illustrating the metabolic profiles in *Panax quinquefolius* L. rhizosphere soil across different continuous cropping treatments. The analysis was based on Euclidean distance, with ellipses representing 95% confidence intervals for each treatment group, indicating the clustering and separation of metabolic profiles.


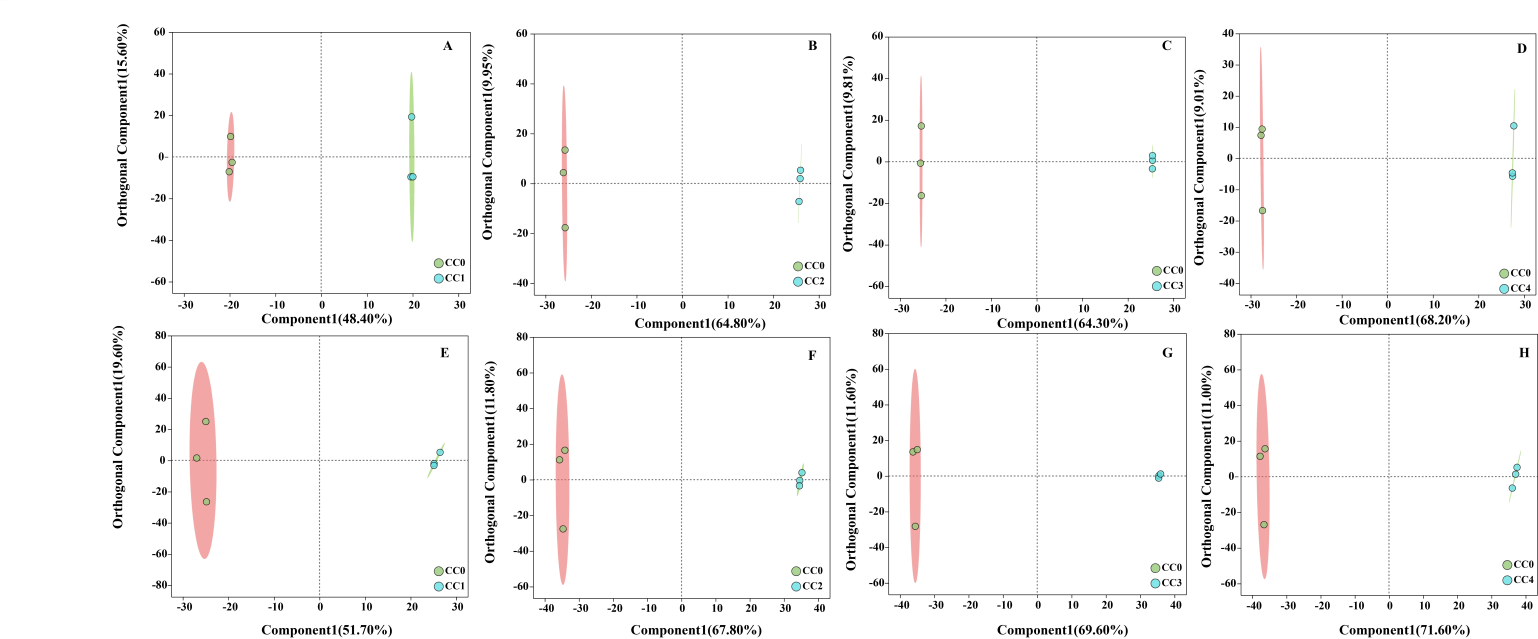


**Fig. S6 Partial Least Squares Discriminant Analysis (PLS-DA) score plots of soil metabolites comparing continuous cropping treatments to control.** Plots show metabolite profiles for (**A**, **E**) CC1 vs. CC0, (**B**, **F**) CC2 vs. CC0, (**C**, **G**) CC3 vs. CC0, and (**D**, **H**) CC4 vs. CC0. Panels (A), (B), (C), and (D) represent positive electrospray ionization modes, while (E), (F), (G), and (H) represent negative electrospray ionization modes. Different colored scatter points correspond to individual soil samples, and ellipses denote the 95% confidence interval for each group, illustrating the separation of metabolic profiles.

**
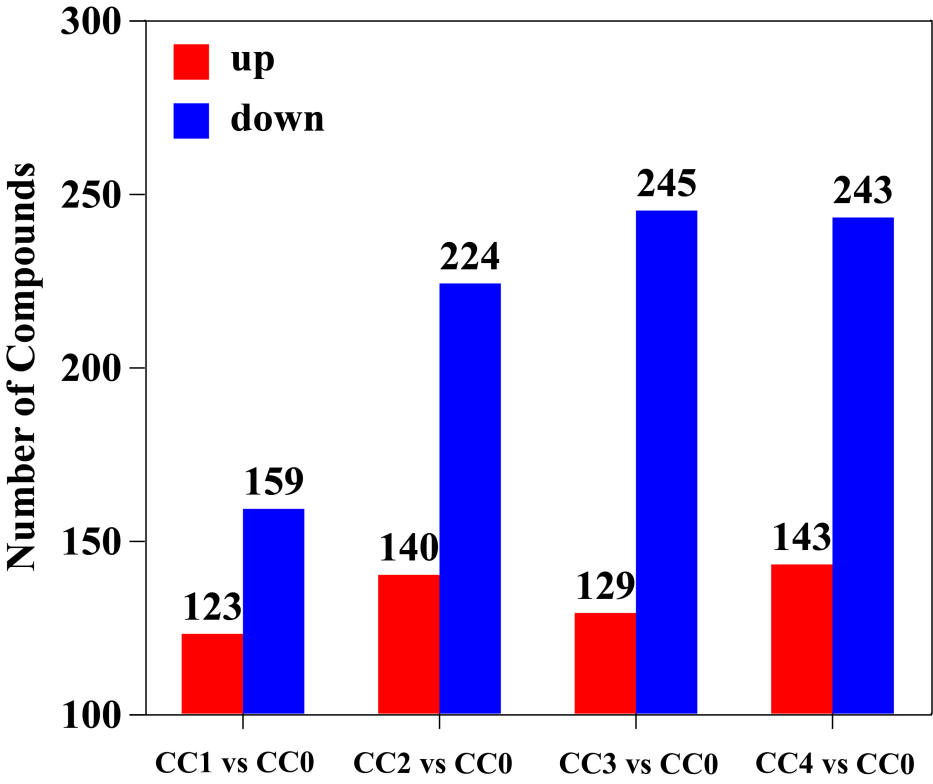
**

**Fig. S7 Number of differentially abundant metabolites (DMs) identified in *Panax quinquefolius* L. rhizosphere soil.** Bar charts show the total number of DMs, as well as upregulated and downregulated metabolites, when comparing (A) 1 year continuous cropping (CC1) vs. control (CC0), (B) 2 years continuous cropping (CC2) vs. control (CC0), (C) 3 years continuous cropping (CC3) vs. control (CC0), and (D) 4 years continuous cropping (CC4) vs. control (CC0).


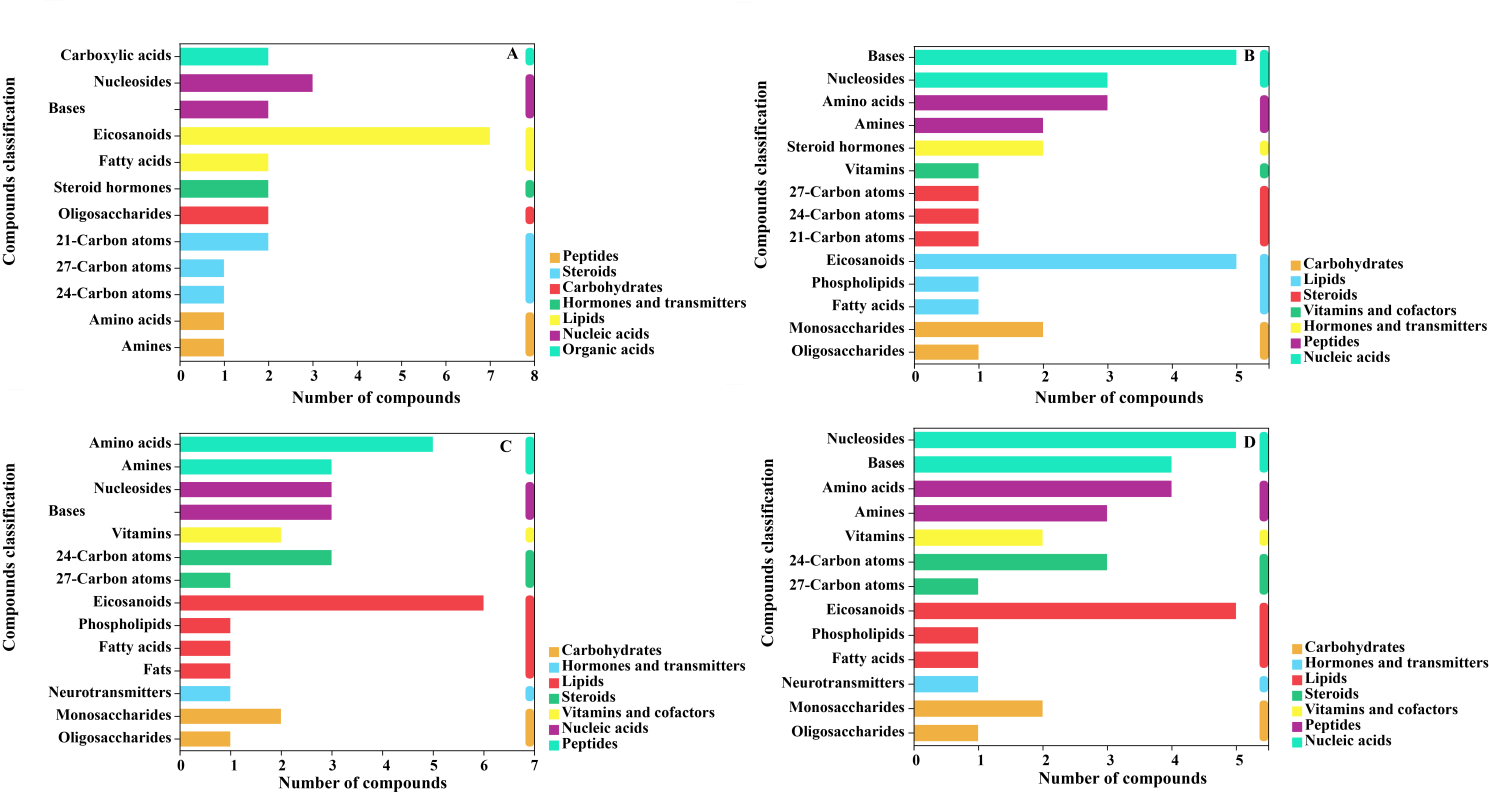


**Fig. S8 Classification of identified differentially abundant metabolites (DMs) based on the KEGG database.** Pie charts illustrate the biochemical categories of DMs when comparing (**A**) 1 year continuous cropping (CC1) vs. control (CC0), (**B**) 2 years continuous cropping (CC2) vs. control (CC0), (**C**) 3 years continuous cropping (CC3) vs. control (CC0), and (**D**) 4 years continuous cropping (CC4) vs. control (CC0).


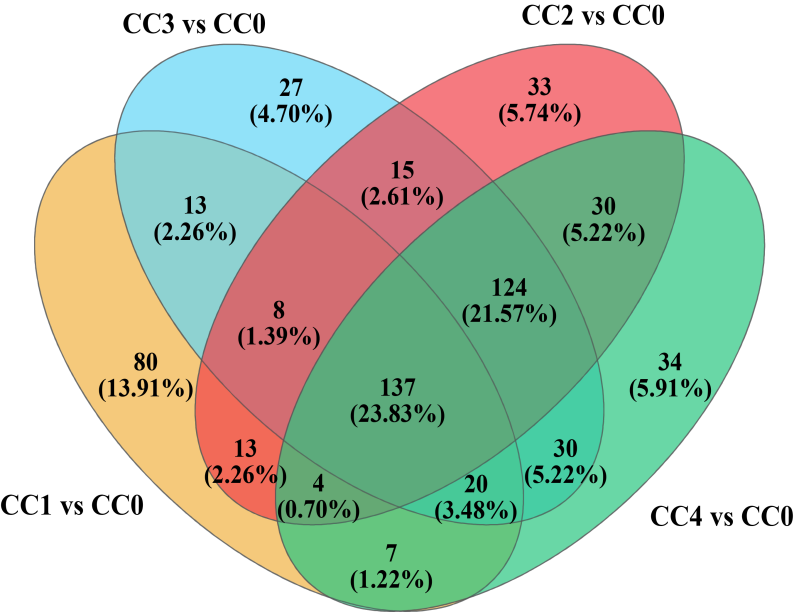


**Fig. S9 Venn diagram illustrating the overlap and uniqueness of differentially abundant metabolites (DMs) across continuous *Panax quinquefolius* L. cropping groups.**


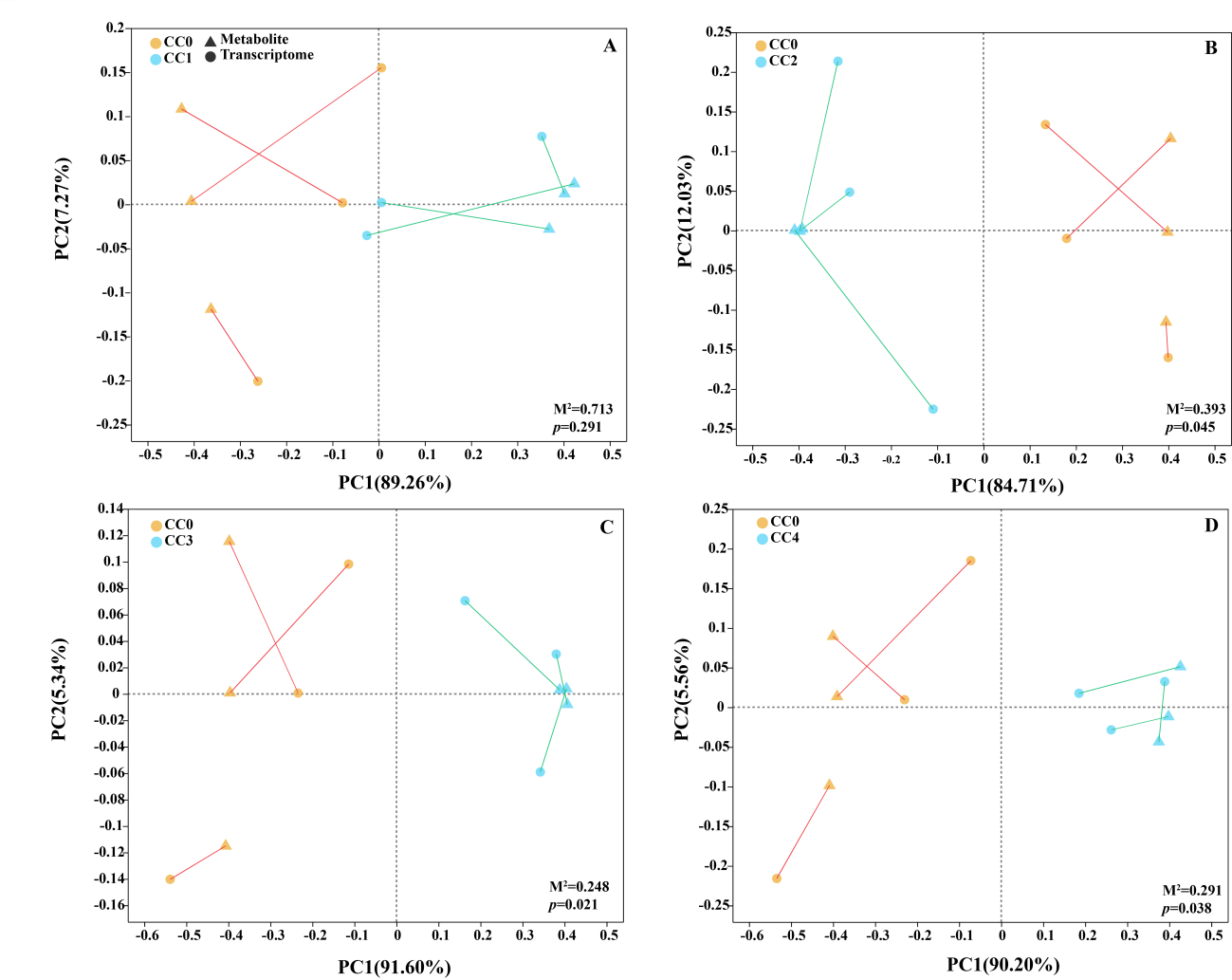


**Fig. S10 Procrustes analysis demonstrating the correlation between soil bacterial community structure and metabolite profiles.** Plots show the relationship between microbial communities and metabolites for (A) CC1 vs. CC0, (B) CC2 vs. CC0, (C) CC3 vs. CC0, and (D) CC4 vs. CC0. M² represents the Procrustes sum of squares, and P indicates the significance level based on 999 permutations. Lines connecting points represent the affine transformation between paired data from the same sample, with shorter lines indicating higher similarity between the microbial community and metabolite profiles.


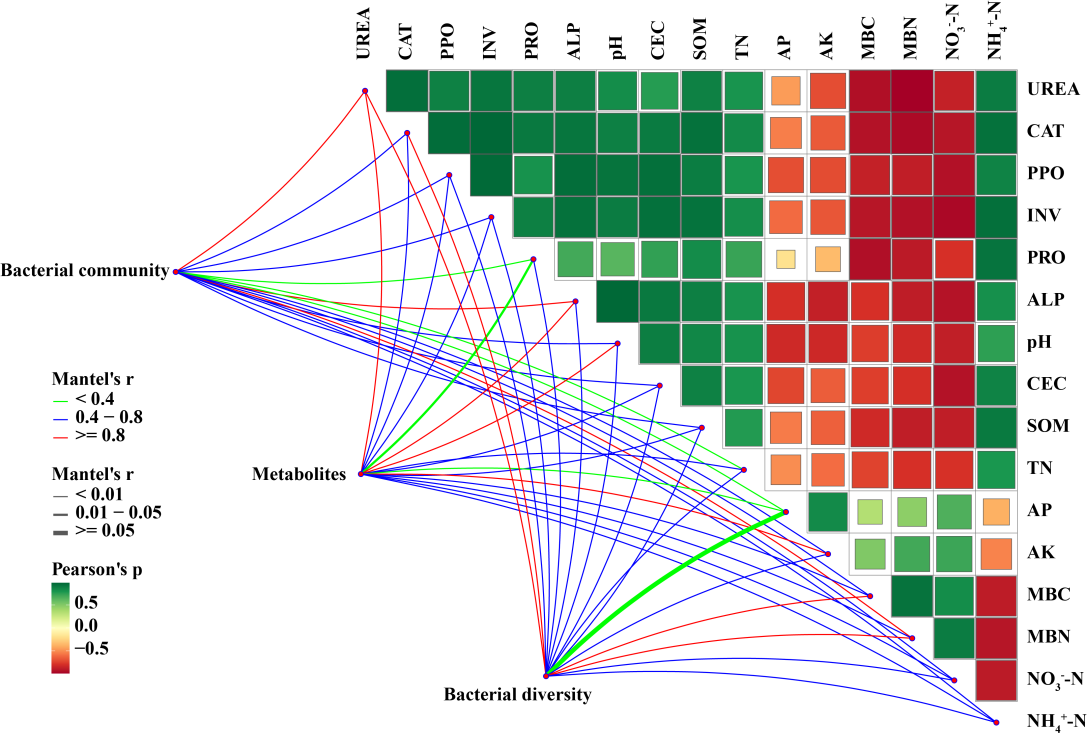


**Fig. S11 Mantel correlations between bacterial community structure, bacterial diversity, metabolite profiles, and various environmental impact factors in continuous *Panax quinquefolius* L. cropping soils.** The heatmap illustrates the correlation coefficients (r-values) between different datasets. AK, soil available potassium; ALP, alkaline phosphatase; AP, soil available phosphorus; CAT, catalase; CEC, cation exchange capacity; INV, invertase; MBC, microbial biomass carbon; MBN, microbial biomass nitrogen; NO_3_^-^-N, nitrate-N; NH_4_^+^, ammonium; PPO, polyphenol oxidase; PRO, protease; SOM, soil organic matter; TN, total nitrogen; UREA, urease.
