# Supplementary material for: Effects of continuous cropping on soil metabolomics and rhizosphere bacterial communities in Panax quinquefolius L
Source: Front Microbiol. 2025 Nov 26;16:1698779. doi: 10.3389/fmicb.2025.1698779 (PMC12689531; doi:10.3389/fmicb.2025.1698779)
Supplement: Supplementary file 2 [file Data_Sheet_2.docx]

| **Table S1 Summary of Illumina MiSeq sequencing and amplicon sequence variants (ASVs) for bacteria.** | | |
| --- | --- | --- |
| Treatments | Sequence number | ASV number |
| CC0-1 | 53105 | 4184 |
| CC0-2 | 52385 | 4568 |
| CC0-3 | 55083 | 4408 |
| CC1-1 | 61486 | 4893 |
| CC1-2 | 56726 | 4455 |
| CC1-3 | 56158 | 4227 |
| CC2-1 | 58244 | 2672 |
| CC2-2 | 60956 | 2431 |
| CC2-3 | 60939 | 2531 |
| CC3-1 | 58756 | 2389 |
| CC3-2 | 57030 | 2408 |
| CC3-3 | 58637 | 2519 |
| CC4-1 | 56781 | 2253 |
| CC4-2 | 57560 | 2373 |
| CC4-3 | 58898 | 2229 |

| **Table S2 The opological indices of co-occurrence network in different continuous *Panax quinquefolius* L. cropping treatments.** | | | | | | | |
| --- | --- | --- | --- | --- | --- | --- | --- |
| Treatments | Nodes | Edges | Ratio of positive edges | Ratio of negative edges | Average weighted degree | Network density | Modularity index |
| CC0 | 199 | 6760 | 54.5% | 45.1% | 67.9 | 0.343 | 0.506 |
| CC1 | 199 | 6618 | 54.0% | 46.0% | 66.5 | 0.336 | 0.555 |
| CC2 | 199 | 6357 | 52.6% | 47.4% | 63.9 | 0.323 | 0.663 |
| CC3 | 199 | 6136 | 50.2% | 49.8% | 61.7 | 0.311 | 0.650 |
| CC4 | 197 | 6065 | 51.9% | 48.1% | 61.6 | 0.314 | 0.640 |

| **Table S3 The contribution of soil environmental factors in affecting the bacterial community.** | | | | |
| --- | --- | --- | --- | --- |
|  | RDA1 | RDA2 | r^2^ | p value |
| SURO | -0.9806 | 0.1959 | 0.5627 | 0.009 |
| NH_4_^+^-N | -1 | 0.0093 | 0.4753 | 0.016 |
| AP | 0.7457 | -0.6663 | 0.5263 | 0.017 |
| ALP | -0.8839 | 0.4678 | 0.7179 | 0.003 |
| TN | -0.8895 | 0.457 | 0.31 | 0.12 |
| NO_3_^-^-N | 0.9837 | -0.18 | 0.5896 | 0.006 |
| UREA | -0.9157 | 0.4019 | 0.5618 | 0.005 |
| CAT | -0.9458 | 0.3246 | 0.5284 | 0.012 |
| pH | -0.8904 | 0.4551 | 0.6439 | 0.005 |
| PPO | -0.9363 | 0.3512 | 0.5153 | 0.017 |
| PRO | -0.9993 | -0.0361 | 0.2663 | 0.158 |
| SOM | -0.9842 | 0.1768 | 0.5423 | 0.009 |
| AK | 0.7102 | -0.704 | 0.8929 | 0.001 |
| CEC | -0.987 | 0.1606 | 0.5136 | 0.022 |
